# Fluorescent protein‐mediated colour polymorphism in reef corals: multicopy genes extend the adaptation/acclimatization potential to variable light environments

**DOI:** 10.1111/mec.13041

**Published:** 2015-01-16

**Authors:** John R. Gittins, Cecilia D'Angelo, Franz Oswald, Richard J. Edwards, Jörg Wiedenmann

**Affiliations:** ^1^ Coral Reef Laboratory, Ocean and Earth ScienceNational Oceanography CentreUniversity of SouthamptonWaterfront CampusSouthamptonSO14 3ZHUK; ^2^ Department of Internal Medicine IUniversity Medical Center Ulm89081UlmGermany; ^3^ School of Biotechnology and Biomolecular SciencesThe University of New South WalesSydneyNSW2052Australia; ^4^ Centre for Biological SciencesUniversity of SouthamptonHighfield CampusSouthamptonSO17 1BJUK; ^5^ Institute for Life SciencesUniversity of SouthamptonHighfield CampusSouthamptonSO17 1BJUK

**Keywords:** acclimatization, adaptation, climate change, copy number variation, coral colour, green fluorescent protein, light regulation, multicopy genes, photoprotection, polymorphism, promoter

## Abstract

The genomic framework that enables corals to adjust to unfavourable conditions is crucial for coral reef survival in a rapidly changing climate. We have explored the striking intraspecific variability in the expression of coral pigments from the green fluorescent protein (GFP) family to elucidate the genomic basis for the plasticity of stress responses among reef corals. We show that multicopy genes can greatly increase the dynamic range over which corals can modulate transcript levels in response to the light environment. Using the red fluorescent protein amilFP597 in the coral *Acropora millepora* as a model, we demonstrate that its expression increases with light intensity, but both the minimal and maximal gene transcript levels vary markedly among colour morphs. The pigment concentration in the tissue of different morphs is strongly correlated with the number of gene copies with a particular promoter type. These findings indicate that colour polymorphism in reef corals can be caused by the environmentally regulated expression of multicopy genes. High‐level expression of amilFP597 is correlated with reduced photodamage of zooxanthellae under acute light stress, supporting a photoprotective function of this pigment. The cluster of light‐regulated pigment genes can enable corals to invest either in expensive high‐level pigmentation, offering benefits under light stress, or to rely on low tissue pigment concentrations and use the conserved resources for other purposes, which is preferable in less light‐exposed environments. The genomic framework described here allows corals to pursue different strategies to succeed in habitats with highly variable light stress levels. In summary, our results suggest that the intraspecific plasticity of reef corals’ stress responses is larger than previously thought.

## Introduction

Shallow‐water coral reefs owe their success in well‐lit tropical waters to the symbiosis of scleractinian corals with dinoflagellates of the genus *Symbiodinium* (zooxanthellae). Solar irradiation in combination with heat and/or nutrient stress causes damage to the resident algal cells that can result in the breakdown of this association and a bleached appearance of the corals (Brown 1997; Warner *et al*. 1999; Douglas 2003; Lesser & Farrell 2004; Baker *et al*. 2008; Wiedenmann *et al*. 2013; D'Angelo & Wiedenmann 2014). Coral mortality caused by mass bleaching contributes to global coral reef decline (Baker *et al*. 2008). Both corals and their symbionts rely on multiple strategies to protect them against heat and light stress in shallow water (Lesser & Shick 1990; Shick *et al*. 1995; Brown *et al*. 2002; Richier *et al*. 2005; Smith *et al*. 2013), which allow some species to survive even in extreme temperature habitats (Hume *et al*. 2013). In *Acropora*, for example, the rapid evolution of proteins responsible for the interaction with the environment seems to promote adaptive processes (Voolstra *et al*. 2011). The adaptive capacity of acroporids is further shaped by distinct expression levels of stress‐response proteins (Barshis *et al*. 2013). Heat stress resistance in *Acropora hyacinthus*, for instance, is associated with high‐level constitutive expression of protective proteins such as heat‐shock proteins and antioxidant enzymes, suggesting that transcriptional frontloading of protective genes may increase stress tolerance (Barshis *et al*. 2013). Striking cases of coral genes displaying strong environmental control are those encoding members of the green fluorescent protein (GFP)‐like pigment family (Alieva *et al*. 2008; D'Angelo *et al*. 2008b, 2012). These pigments are responsible for most of the conspicuous green, red and purple‐blue coloration of hermatypic reef corals and several other cnidarians (Dove *et al*. 2001; Alieva *et al*. 2008) and can constitute up to 14% of the total soluble proteins in the expressing tissue (Leutenegger *et al*. 2007b; Oswald *et al*. 2007; Smith *et al*. 2013). As their presence in coral tissue can be detected and quantified by noninvasive optical techniques in situ and in vivo, they represent ideal candidates for studies of environmentally regulated genes (D'Angelo *et al*. 2008b, 2012). Some coral species can express an array of different fluorescent proteins (FPs) and brightly coloured, but nonfluorescent chromoproteins (CPs) (Kelmanson & Matz 2003; Oswald *et al*. 2007; Alieva *et al*. 2008; D'Angelo *et al*. 2008b). For example, a single individual of *Acropora millepora* was shown to express three different cyan and green FPs, three spectrally distinct purple‐blue CPs and one red FP (amilFP597) (D'Angelo *et al*. 2008b; Smith *et al*. 2013). Visibly distinct colour morphs of anthozoans in which one or a few pigments are accumulated in high amounts can result from stable differences in the FP and CP expression levels (Kelmanson & Matz 2003; Leutenegger *et al*. 2007a; Oswald *et al*. 2007; Schnitzler *et al*. 2008; Smith *et al*. 2013).

**Figure 1:**
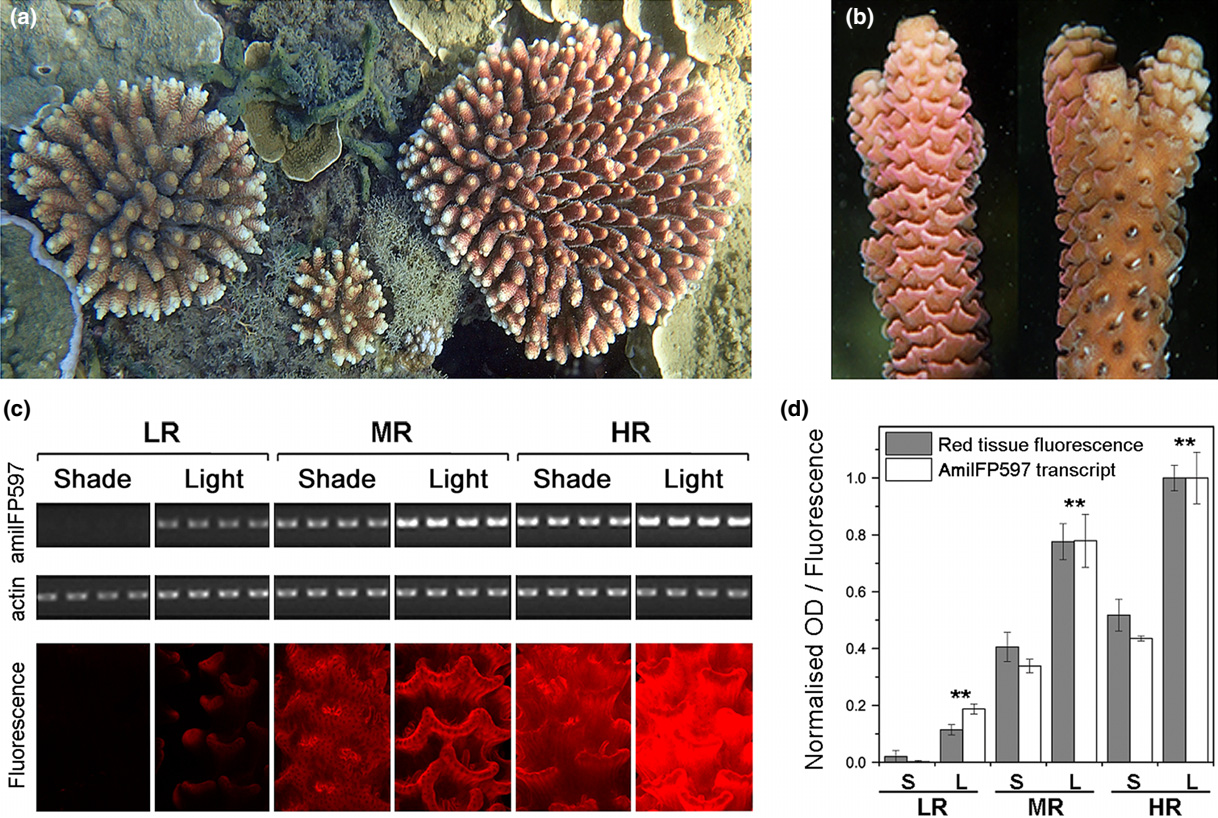
Colour polymorphism of *Acropora millepora*. (a) Different red fluorescent protein (RFP) levels accumulated by *A. millepora* colour morphs in response to identical environmental conditions in Florence Bay, Magnetic Island, Great Barrier Reef, Australia. (b) Representative images show the increased RFP content in the light‐exposed branch side (left) compared to the shaded branch side (right). (c) Lower panel: Fluorescence micrographs of three colour morphs (LR, low red; MR, medium red; HR, high red) cultured in the laboratory under the same light intensity showing different RFP (amilFP597) contents in the upper (Light) and lower (Shade) branch sides. Upper panel: Semi‐quantitative RT‐PCR analyses of RFP expression in the respective branch sides of LR, MR and HR morphs. Ethidium bromide‐stained agarose gels show the RFP and actin amplicons. (d) Mean RFP transcript levels normalized to the actin control in shaded (S) and light‐exposed (L) branch sides. Error bars denote standard deviation. Asterisks indicate significant differences (anova;* P* < 0.01) among samples from shaded and light‐exposed branch sides, respectively.

The production of GFP‐like proteins in anthozoans from shallow‐water habitats has been suggested as a host strategy to protect the symbiotic algae from high irradiances by absorbing photons or distributing them away from the algal pigments by re‐emission or reflectance (Wiedenmann *et al*. 1999; Salih *et al*. 2000; Dove *et al*. 2001; D'Angelo *et al*. 2008b, 2012). Screening of zooxanthellae from photosynthetically active radiat‐ion by high CP contents was demonstrated for colour morphs of several coral species and was associated with a reduction of photodamage and resulting loss of zooxanthellae (bleaching) under experimental light stress (Smith *et al*. 2013). CP‐mediated screening is also thought to facilitate the colonization of growing or regenerating tissue with zooxanthellae (D'Angelo *et al*. 2012; Smith *et al*. 2013) and to modulate symbiont photosynthesis (Dove *et al*. 2008). Consistent with their proposed photoprotective function, the expression of GFP‐like proteins in many shallow‐water corals is regulated by light intensity, particularly in the blue spectral range (D'Angelo *et al*. 2008b, 2012; Smith *et al*. 2013). Their expression can be negatively affected by heat, which may potentially render some corals more vulnerable to light stress during episodes of elevated temperatures (Smith‐Keune & Dove 2007; D'Angelo *et al*. 2008a; Hume *et al*. 2013). Stress induced by the corallivorous flatworm *Amakusaplana acroporae* can also result in a reduction of the FP content in the tissue of infested colonies (Hume *et al*. 2014). Highly pigmented colour morphs of certain anthozoan species including corals are frequently found in the shallowest sites of their habitats, suggesting a selective advantage of pigmentation in high‐light environments (Wiedenmann *et al*. 1999, 2007; Salih *et al*. 2000, 2006; Smith *et al*. 2013). However, colour morphs often display an overlapping distribution (Wiedenmann *et al*. 1999; Kelmanson & Matz 2003; Salih *et al*. 2006; Leutenegger *et al*. 2007a), and conspecific corals containing widely different concentrations of the same types of GFP‐like proteins can be found side by side in the most light‐exposed reef sites (Figs 1a and S1, Supporting information). This coexistence suggests that the benefits afforded by these pigments are not essential, but that they offer more subtle advantages, such as enabling corals to acclimate to a broader range of conditions or to survive extreme stress events (Smith *et al*. 2013). The presence of GFP‐like proteins in corals from low‐light habitats as well as in azooxanthellate and deep sea anthozoans suggests that these proteins fulfil a range of functions apart from photoprotection (Salih *et al*. 2000; Wiedenmann *et al*. 2004a; Leutenegger *et al*. 2007b; Dove *et al*. 2008; Vogt *et al*. 2008).

Variation in gene copy number, first detected among human populations, can result in differential gene expression and, consequently, in distinct phenotypic traits (Korbel *et al*. 2008). The existence of copy number polymorphisms might significantly shorten the evolutionary time required for adaptation to environmental conditions that demand altered expression of certain genes (Perry *et al*. 2007; Korbel *et al*. 2008). To understand the plasticity of corals’ responses to environmental stimuli and to elucidate the genomic basis for large differences in the constitutive expression of environmentally regulated genes, we have explored whether copy number variations are responsible for the different expression levels of GFP‐like pigments in colour morphs of *A. millepora*. The colour morphs of this species show a range of cyan‐green, red and purple‐blue hues caused by GFP‐like proteins (D'Angelo *et al*. 2008b; D'Angelo & Wiedenmann 2011; Smith *et al*. 2013). Among them, the red FP amilFP597 is ideally suited for the analysis of the genomic basis of the intraspecific colour variability as it (i) is defined by a distinct nucleotide/amino acid sequence, (ii) displays a characteristic fluorescence signature that permits its optical quantification even in complex FP mixtures and (iii) shows a uniform upregulation in response to increased light levels (D'Angelo *et al*. 2008b).

## Materials and methods

### Coral culture and light stress experiments

*Acropora millepora* morphs originating from Fijian reefs were acquired through the Tropical Marine Centre (London, UK) (D'Angelo & Wiedenmann 2012). We focused on three colonies representing morphs with distinct levels of redness: high‐level red (HR), medium‐level red (MR) and low‐level red (LR). Additionally, individuals of three other morphs with intermediate levels of redness (IR1, IR2) and nearly undetectable redness (NR) were also used for parts of this study. The IR1 morph represents a previously characterized red morph (D'Angelo *et al*. 2008b). The mother colonies were fragmented and regrown into replicate colonies. Minimal acclimatization times required by the corals to adjust their tissue pigment levels to a given light environment were determined as detailed in Supporting Information and in Fig. S2. Subsequently, >5 replicate colonies of the different colour morphs were cultured side by side in tanks for at least 6 months, exposed to identical photon fluxes of 300 μmol/m^2^/s provided by 250‐W metal halide lamps (Aqualine 10000, 13000 K; Aqua Medic, Germany) with a 12‐h light:dark cycle. The water temperature was maintained at a constant 25 ± 0.5 °C. The experimental set‐up was as described previously (D'Angelo & Wiedenmann 2012).

Focused exposure of replicate branches of the HR and LR morphs to yellow‐orange light (500 μmol/m^2^/s) was achieved by mounting a MZ10 fluorescence stereomicroscope (Leica Microsystems, Germany) equipped with a 550–596‐nm bandpass filter (AHF, Germany) over a temperature‐controlled flow‐through compartment. Corals were exposed to light in a 10‐h light: 14‐h dark cycle. After three light cycles, the maximum quantum yield (*F*_v_/*F*_m_) of zooxanthellae photosynthesis after a 14‐h dark recovery period was measured using a Diving PAM (Waltz, Germany), as described previously (Smith *et al*. 2013).

### Red fluorescent protein expression in different coral colour morphs

Branches of the LR, MR and HR morphs of *A. millepora* were sampled and their upper‐ and undersides were photographed under a fluorescence stereomicroscope (Leica MZ10F) with a dsRED filter set (excitation band 525–580 nm, emission band 590–690 nm; AHF), using the same magnification and identical exposure times. Red channel images were used for quantitative analysis. Tissue fluorescence of the different morphs was also determined using a Cary Eclipse fluorescence spectrophotometer (Varian, USA) with a fibre optic probe, as described previously (D'Angelo *et al*. 2008b, 2012). Tissue was sampled from the upper branch sides of the LR and HR morphs and homogenized with a micropestle in phosphate‐buffered saline (pH 7.8). The homogenate was centrifuged for 1 h at 4 °C/20 000 ***g***. The absorption spectra of the clarified supernatants were recorded in a Cary^®^ 50 UV‐Vis spectrophotometer (Varian), background‐corrected and normalized to the absorption values at 280 nm (aromatic amino acid absorption).

Total RNA was prepared from the tissues of light‐exposed and shaded branch sides as described previously (D'Angelo *et al*. 2008b, 2012). Identical amounts of RNA (500 ng/sample) were reverse transcribed, and the resulting cDNA was used as the template in semi‐quantitative PCR to quantify *amilFP597* transcript levels as described before (D'Angelo *et al*. 2008b, 2012). Actin transcript levels, determined in parallel reactions using primers specific for coral actin, were used to normalize the *amilFP597* data. First, control PCRs were performed to ensure that all semi‐quantitative analyses were conducted in the exponential phase of amplification (D'Angelo *et al*. 2008b, 2012). Four independent PCRs were analysed per transcript type and colony. Band volumes (intensity units × mm^2^) of PCR products separated on agarose gels (2%) containing ethidium bromide were quantified using quantity one (Bio‐Rad) image analysis software. Oligonucleotide primers utilized for PCR are detailed in Table S1 (Supporting information).

### Analysis of *amilFP597* gene structure and copy number

Genomic DNA was purified from six different *A. millepora* morphs (HR, MR, LR, NR, IR1, IR2) as previou‐sly described (Sokolov 2000) (proteinase K treatment omitted). The integrity of the DNA preparation was confirmed by agarose gel electrophoresis, and the concentration was accurately quantified using a dye‐binding fluorescence assay (DNA Quantification Kit; Sigma, USA). PCR amplification with Advantage^®^ 2 polymerase mix (Clontech, USA), cloning (StrataClone PCR Cloning Kit; Agilent Technologies, USA) and sequencing (Macrogen Europe, the Netherlands) were applied throughout this study.

Initially, the GenomeWalker Universal Kit (Clontech) was used to obtain a 2.1‐kb fragment of the MR morph genome extending from exon 2 of *amilFP597* to the putative promoter region of this gene (GenBank Accession no. JQ009183). In addition, the 5′ and 3′ untranslated regions (UTRs) of the *amilFP597* transcript were determined using a SMART RACE kit (Clontech).

Full‐length sequences of *indel (+)* and *indel (−)* promoter variants of the *amilFP597* gene were obtained for the MR morph. These sequences, extending from the promoter region to the 3′UTR, were produced by joining two overlapping PCR products covering the 5′ region (promoter to exon 3) and the 3′ region (intron 2 to 3′UTR). The 5′ region fragments were amplified from MR morph genomic DNA using forward primers specific for the *indel (+)* and *indel (−)* variant promoters in combination with a primer used in the primary GenomeWalker reactions. The 3′ region fragments were amplified from the same DNA template using a forward primer in intron 2 and a reverse primer in the 3′UTR. Sequence differences in the overlap between the 5′ and 3′ region fragments were used to assign the latter to either the *indel (+)* or *indel (−)* promoter variant genes. These reconstructed gene sequences have been submitted to GenBank as Accessions KC818413 [*indel (−)* gene] and KC818414 [*indel (+)* gene].

DNA fragments (~3 kb) linking *amilFP597* gene copies within tandem arrays were amplified by PCR, cloned and sequenced as described in the Supporting Information.

To quantify the abundance of *amilFP597* and related variant genes among the LR, MR and HR morphs, primers designed to conserved regions of exon 3 (RFPex3consF/RFPex3consR) were used to amplify a 155‐bp fragment spanning the chromophore coding sequence from identical amounts of genomic DNA in a semi‐quantitative PCR using Advantage^®^ 2 polymerase mix. After 26 thermocycles, in the exponential phase of the amplification process (Fig. S3, Supporting information), the products were separated on ethidium bromide agarose gels (2%) and the band volumes were quantified as described above. For each colour morph, the experiment was performed four times, with each repetition featuring triplicate reactions, and means with standard deviation were calculated. The results were verified using two separate genomic DNA preparations for each colour morph.

In addition, products from the triplicate PCRs were pooled and digested with *Ape*KI, a restriction enzyme that specifically cleaves *amilFP597* exon 3 fragment sequences. The restriction products and undigested samples were separated on ethidium bromide agarose gels (3%), and the proportion of exon 3 fragments digested by *Ape*KI was quantified as before. The experiment was performed four times, and the abundance of *amilFP597* copies in each morph was calculated from the average percentage of *Ape*KI‐digested exon 3 fragments. The comparable frequency of *amilFP597* copies among the different *A. millepora* morphs was confirmed by sequencing ~65 randomly selected exon 3 fragment clones per morph. Differences in the detection frequency of *amilFP597* and related variant genes among the panel of exon 3 fragment clones were also used to estimate the copy number of the former. In a control experiment, we confirmed that the PCR amplification characteristics of the 155‐bp exon 3 fragments and the cloning efficiency of the resulting amplicons are identical for different *amilFP597* variants and suitable for semi‐quantitative comparison (Supporting Information and Fig. S3).

Oligonucleotide primers used for these analyses are detailed in Table S1 (Supporting information).

### Diversity of *amilFP597*‐related genes and characterization of variant proteins

To examine the diversity of *amilFP597*‐related genes in the HR, MR and LR morphs, ~65 sequences of the 155‐bp fragments amplified using consensus exon 3 primers as described above were aligned for each morph and grouped according to sequence homology. Only sequence mismatches that were found at least twice (or whose authenticity was confirmed by their detection in independent cDNA or genomic DNA sequences) were included in the analysis to filter out potential PCR/sequencing errors. Besides amilFP597, these exon 3 fragments encoded four other hypothetical amilFP597‐related protein variants, three of which possess different chromophore types. Their sequences showed high similarity to an *A. millepora* larval mRNA encoding an amilFP597‐variant with a TYG chromophore (GenBank Accession no. EZ013771; Meyer *et al*. 2009). DNA fragments comprising the open reading frames (ORFs) of two variants (amilCP506, amilCP564) were amplified from cDNA generated from the MR morph as described previously (D'Angelo *et al*. 2008b), using primers (14_28cDNA_F/14_28cDNA_R) designed against the sequence of the larval transcript. Aliquots of the PCRs were examined by ethidium bromide agarose gel (0.8%) electrophoresis after 30 and 35 amplification cycles. An identical PCR using the same cDNA template with *amilFP597* ORF primers (14_28cDNA_F/AmRFPRev) was performed in parallel for semi‐quantitative comparison of transcript levels in adult *A. millepora*. The cDNA encoding another amilFP597 variant A1a (GenBank Accession no. AAT77753.1; (Smith‐Keune & Dove 2007) was re‐created by PCR mutagenesis (Kredel *et al*. 2009), introducing the amino acid exchanges N34D and M44I into amilFP597. The synthetic ORF sequence has been deposited in GenBank as Accession no. KJ729554.

Three variant cDNAs (*amilCP506*,* amilCP564*,* A1a*) were cloned in the vector pQE30 (Qiagen, Germany), expressed in *Escherichia coli* XL1‐Blue (Wiedenmann *et al*. 2002) and purified by immobilized metal ion chromatography using Talon^®^ resin (Clontech). Following removal of imidazole from the samples by ultrafiltration (Amicon Ultra‐15; Merck Millipore, USA), the spectral properties of the red fluorescent protein (RFP)‐related proteins (absorption and fluorescence spectra, molar extinction coefficient) were analysed as described previously (Kredel *et al*. 2008). Fluorescence quantum yields were determined relative to the green form of EosFP and to eqFP611 (Wiedenmann *et al*. 2002, 2004b). In agreement with the nomenclature for FPs (D'Angelo *et al*. 2008b), the amilFP597 variant encoded by the A1a transcript should be named amilFP605.

### Molecular phylogenetic analyses

Molecular phylogenetic analyses of GFP‐like protein sequences were conducted using the mega5 software package (Tamura *et al*. 2011). A multiple sequence alignment of GFP‐like proteins (Alieva *et al*. 2008; courtesy of the Matz lab, University of Texas) was extended using sequences of GFP‐like proteins from *A. millepora* isolated during this study and in previous projects (D'Angelo *et al*. 2008b; Smith *et al*. 2013). The evolutionary history was inferred using the maximum‐likelihood method. The JTT matrix‐based model (Jones *et al*. 1992) and the Tamura–Nei model (Tamura & Nei 1993) were used for protein and nucleotide sequence data, respectively. The tree with the highest log likelihood was chosen. Initial trees for the heuristic search were obtained automatically by applying the Neighbor‐Join and BioNJ algorithms to a matrix of pairwise distances and then selecting the topology with the superior log‐likelihood value. All trees were drawn to scale and mid‐point rooted, with branch lengths measured in the number of substitutions per site. All positions containing gaps and missing data were eliminated. Branch confidence was assessed using 100 bootstrap replicates.

### *amilFP597* promoter variability

The putative promoter region of the *amilFP597* gene, extending ~1.1 kb upstream of the start codon, was examined in six *A. millepora* colour morphs by amplifying this region using the primers AmRFPp‐F3/AmRFPp‐R1 in standardized PCRs. This primer pair is specific for the *amilFP597* genes and does not amplify promoter fragments from genes encoding the paralogous variants (amilCP506, amilCP564, vRFP3, vRFP4). The amplified fragments were cloned and at least five clones per morph sequenced. Multiple alignments of the sequences using clustalw2 (http://www.ebi.ac.uk/Tools/msa/clustalw2/) and MegAlign (DNAstar Lasergene 9) demonstrated the presence of *indel (+)* and *indel (−)* promoter variants. The *indel (+)* form was found in all morphs, and the second indel I2 contained a diagnostic *Hpa*I restriction site (GTTAAC). To determine whether the tissue concentration of amilFP597 correlated with the proportion of *amilFP597* copies associated with the two promoter variants, 20 randomly selected 1.1‐kb promoter fragment clones for each morph were digested with *Hpa*I. To verify these results by an alternative method, primers were designed to amplify fragments specifically from the *indel (+)* and *indel (−)* forms (pRFPlargeF and pRFPsmallF, respectively). These 27‐mers were identical apart from the four nucleotides comprising their 3′ termini: in the primer pRFPlargeF [*indel (+)* variant], the 3′ end sequence was the first four nucleotides of indel I1 (TCAC), while in pRFPsmallF [*indel (−)* variant], it was the four nucleotides immediately following indel I1 (GTCT). These primers were used in combination with the common reverse primer AmRFPp‐R1 in separate semi‐quantitative PCRs. We confirmed that these primer pairs do not amplify the paralogous a*milFP597* variants and their promoters. The amplified ~833‐ and 796 bp‐fragments from the *indel (+)* and *indel (−)* forms, respectively, were separated on ethidium bromide agarose gels (2%) and quantified to assess the abundance of each form, as described above. The activity of the *indel (+)* and *indel (−) amilFP597* promoter variants was analysed in a heterologous luciferase reporter gene assay (Oswald *et al*. 2002) as detailed in Supporting Information.

### Statistical analysis

The significance of the differences in transcript levels observed among the three morphs exposed to identical light environments was calculated with one‐way anova followed by Bonferroni correction for multiple comparisons. The same test was applied to determine the significance of differences in PCR amplification efficiency of paralogous *amilFP597* variants (Supporting Information and Fig. S3). All analyses were performed using instat (GraphPad software, USA). Curve fits (Figs 4, S2 and S3, Supporting information) were calculated with Origin (OriginLab Corp, USA). To further test the statistical strength of the linear relationship between the percentage of *indel (−) amilFP597* genes, determined using the two different methods (restriction analysis of amplified promoter fragments and semi‐quantitative PCR), and the red fluorescence of the *A. millepora* colonies measured in vivo, we used mixed effects models fitted using maximum likelihood (ML), conducted in the package ‘nlme’ (Pinheiro *et al*. 2014). We included the random effect of genotype, which was repeatedly assessed for each method. The model fit and residual structure were visually inspected to ensure that the test assumptions were met (graphical residual analysis; Crawley 2012). Contrast coefficient estimates for fixed effects are reported as treatment contrasts (Type I sum of squares) in the model results summary (Table 1).

**Table 1:** Result summary of the mixed effect model analysis

Fixed effects	Estimate	SE	*t*‐value	*P*‐value
Intercept	4.143	10.301	0.402	0.704
% *indel (−)* genes	1.112	0.238	4.662	0.0186
Restriction analysis	1.332	9.0977	0.146	0.893
Restriction: semi‐quantitative PCR	−0.055	0.212	−0.260	0.811

## Results and discussion

We studied colour polymorphisms of *Acropora millepora* to understand the plasticity of corals’ responses to environmental stimuli and to elucidate the genomic basis for large differences in the constitutive expression of environmentally regulated genes.

Colour morphs of *A. millepora* growing under the same light levels in shallow water in the Great Barrier Reef can differ considerably in their degree of redness (Figs 1a and S1, Supporting information). The light‐driven upregulation of RFPs responsible for the red pigmentation of *A*. *millepora*, demonstrated previously in laboratory experiments (D'Angelo *et al*. 2008b), is also evident from the increased red pigmentation of light‐exposed parts of branches (Figs 1b–d and S2, Supporting information). However, this light regulation does not explain the pronounced differences in RFP‐mediated pigmentation in different morphs exposed to the same light intensity (Figs 1a and S1, Supporting information).

Based on quantitative and qualitative analysis of the RFP content, we selected representatives of *A. millepora* that differ in their degree of redness to explore the genomic basis of colour polymorphism. We initially focused on high‐level red (HR), MR and LR morphs. A time‐course measurement of tissue fluorescence revealed that *A. millepora* takes up to 6 weeks to acclimatize to increased or reduced light levels (Supporting Information and Fig. S2). Therefore, corals were cultured side by side for more than 6 months in our experimental mesocosm (D'Angelo & Wiedenmann 2012) under identical light levels to allow sufficient time for the RFP pigment concentration in the tissue to reach the characteristic values for the specific light environment. Spectroscopic analysis of the morphs showed that their red tissue fluorescence peaks at around 597 nm (Fig. S4, Supporting information). In agreement with our field observations (Fig. 1b), the red fluorescence in the upper branch surfaces of all morphs was considerably brighter than that of the shaded side (Fig. 1c,d). The differences in maximal fluorescence intensity among the morphs were also seen in the values determined for their shaded branch sides.

Cloning and sequencing of the RFP‐encoding cDNAs identified amilFP597 (D'Angelo *et al*. 2008b) as the protein responsible for the red fluorescence signal in all morphs. The highly similar transcript A1a, previously identified in an *A. millepora* colony from the Great Barrier Reef (Smith‐Keune & Dove 2007), was not detected. Semi‐quantitative RT‐PCR showed that *amilFP597* transcript levels correlate closely with the measured differences in red tissue fluorescence between the shaded and light‐exposed tissues and between the different colour morphs (Fig. 1c,d). Importantly, the constitutive amilFP597 expression in the shaded branch sides of the HR morph exceeds the upregulated expression in the light‐exposed areas of the LR morph. Therefore, variation in the red tissue fluorescence of *A. millepora* can be considered a direct result of different *amilFP597* transcript levels.

### Structure of the *amilFP597* gene

**Figure 2:**
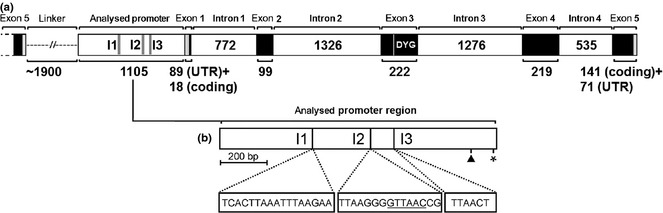
Schematic representation of the *amilFP597* gene within a gene tandem array. (a) The five exons of the gene are displayed as black bars with their length in nucleotides indicated beneath brackets. The grey‐shaded regions in exons 1 and 5 correspond to the 5′ and 3′UTRs, respectively. The chromophore‐forming amino acid triplet (DYG) is located within exon 3 and is marked by a white vertical line. The four introns are shown as white bars with their respective sizes. The analysed promoter region of the gene extends 1105 bp upstream of the 5′UTR. The positions of three indels (I1–I3) differentiating the *indel (+)* and the *indel (−)* promoter variants of *amilFP597* are indicated in the promoter region. The DNA fragment linking a putative first *amilFP597* copy with the analysed promoter region of the downstream copy is depicted by a dashed line. (b) Schematic representation of the analysed *amilFP597* promoter region. The putative transcription initiation site (arrowhead) and the start codon (asterisk) are marked. The positions of the indel sequences (I1–I3) are marked by dotted lines, and their sequences are shown in boxes below the promoter diagram. The *Hpa*I site in indel 2 used for restriction analysis is underlined.

We used a PCR‐based strategy to reconstruct the *amilFP597* gene. This gene consists of the coding region interspersed by four introns and flanked by untranslated regions (UTRs) (Fig. 2a). A ~1.1‐kb fragment upstream of the start codon demonstrated strong promoter activity in heterologous luciferase reporter gene assays (Fig. S5, Supporting information). Sequence analysis of separate PCR‐amplified promoter fragment clones demonstrated the presence of two highly conserved variants that are distinguished by three short indels. These variants were designated *indel (+)* and *indel (−)* for the long and short form, respectively (Figs 2b and S6, Supporting information). *A. millepora* morphs expressing amilFP597 only at very low levels (LR, NR) contained only the *indel (+)* variant of *amilFP597*, whereas the four other morphs contained a mixture of both forms of this gene. We used a PCR approach to determine whether *amilFP597* genes are found in tandem arrays like those of CP‐encoding genes in *A. millepora* (Beltran Ramirez 2010). The specific primer combination used for this experiment was designed to yield a product only when the *amilFP597* copies are arranged in head to tail tandems. Using nested primers binding in exon 5 (forward) and the proximal promoter region (reverse) of *amilFP*597, we amplified a dominant fragment of ~3 kb from the LR, MR and HR morphs. Cloning and sequencing of this linker from the MR morph revealed two separate forms with 98.2% nucleotide sequence identity, containing the *indel (+)* and *indel (−)* promoter variants, respectively (GenBank Accession nos. KM101115 and KM101116). This result clearly demonstrates that *amilFP597* genes can occur in head‐to‐tail tandem arrangements in the *A. millepora* genome. As tandem gene duplication is a major driver of gene multiplication in eukaryotes (Zhang 2003) and the putative reason for clustering of genes encoding cyan and green FPs in the genome of *Acropora digitifera* (Shinzato *et al*. 2012), this mechanism is presumably responsible for the multiple *amilFP597* copies in the *A. millepora* genome.

### Copy number variations of the *indel (−)* promoter form of *amilFP597* are correlated with morph‐specific RFP expression

To assess the intraspecific variability of *amilFP597* and related genes, we first amplified exon 3 fragments (155 bp) spanning the chromophore‐coding region using genomic DNA from different *A. millepora* colour morphs as template. Cloning and sequencing of these fragments revealed genes encoding four hypothetical amilFP597‐related proteins, including three forms possessing different chromophore types (Fig. S7, Supporting information). DNA fragments comprising the ORFs of two distinct *amilFP597* variants were amplified from cDNA generated from the MR morph (D'Angelo *et al*. 2008b) using primers designed against the sequence of a larval transcript (Meyer *et al*. 2009) in a semi‐quantitative PCR. After cloning and recombinant expression, biochemical characterization of the encoded proteins revealed that these two variants represent unusual green and purple CPs, which we have named amilCP506 and amilCP564 (Supporting Information and Fig. S8).

We detected transcripts of these variants at very low levels in adult *A. millepora* colonies (>100× less frequent than amilFP597 transcripts, Fig. S8, Supporting information). In contrast, these variants are expressed at high level in *A. millepora* larvae (Beltran Ramirez 2010).

Molecular phylogenetic comparison with the CPs amilCP575, amilCP584 and amilCP604 expressed in some adult *A. millepora* morphs (Smith *et al*. 2013) clearly indicated that amilCP506 and amilCP564 are directly derived from amilFP597 and that they have gained their excellent screening properties (Table S2, Supporting information) in a parallel evolution process (Fig. S8, Supporting information).

Among the panel of characterized 155‐bp exon 3 fragment clones, those representing *amilFP597* were found around eight times more frequently than the least abundant gene variant (vRFP3; Fig. S7, Supporting information). As the latter must represent at least a single copy gene, this suggests that *amilFP597* is present in multiple (≥8) copies in the *A. millepora* genome.

**Figure 3:**
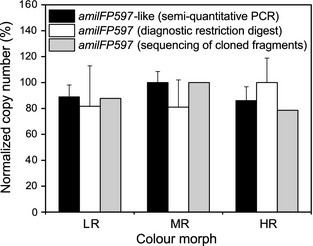
Normalized numbers of *amilFP597* copies in the LR, MR and HR morphs of *Acropora millepora*. Copy numbers of *amilFP597* variant genes were determined by semi‐quantitative PCR amplification of a conserved genomic exon 3 fragment. The *amilFP597* copy numbers were independently determined by diagnostic restriction analysis with *Ape*KI and sequencing of ~65 randomly selected exon 3 fragments cloned from each of the morphs. The graph shows the mean values and standard deviations. To normalize the data, the maximal value obtained from each experimental approach was set to 100% and the corresponding values for the different morphs were scaled accordingly. HR, high‐level red; LR, low‐level red; MR, medium‐level red.

A straightforward explanation for the observed variability in RFP levels in the studied *A. millepora* morphs could be offered by differences in the number of *amilFP597* copies. However, semi‐quantitative PCR analyses in combination with diagnostic restriction digests and sequencing of the amplified exon 3 fragments revealed no variation in the absolute *amilFP597* copy number that correlates with the characteristic RFP levels in the separate morphs (Fig. 3). As clonal replicates of only three genotypes/morphs were used in these analyses, the interpretability of the results (Figs 1d and 3) is limited.

**Figure 4:**
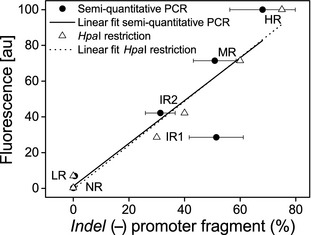
Analysis of the *amilFP597* promoter in six *Acropora millepora* morphs with different levels of redness, including the HR, MR and LR morphs. Graph showing the amilFP597 tissue content of different morphs growing under the same light intensity plotted against the relative contribution of the *indel (−)* promoter type to the total *amilFP597* copy number in the respective morphs. Promoter copy ratios were evaluated independently by diagnostic restriction analysis of amplified and cloned promoter fragments using *Hpa*I (triangles) and by semi‐quantitative PCR (filled spheres represent mean values, error bars denote standard deviation). The linear regressions for the two data sets are essentially identical and statistically significant at a >98% confidence level (restriction analysis: *R* ^2^ = 0.97/Adjusted *R* ^2^ = 0.96/*P* = 0.00038; semi‐quantitative PCR:* R* ^2^ = 0.78/Adjusted *R* ^2^ = 0.73/*P* = 0.01901). HR, high‐level red; LR, low‐level red; MR, medium‐level red.

Therefore, we determined the ratios of the *indel (+)* and *indel (−) amilFP597* genes in the LR, MR and HR morphs along with three additional morphs to examine whether sequence divergence in the regulatory region may be responsible for differential RFP expression (Fig. 4).

These findings indicate that the pronounced differences in the minimal and maximal *amilFP597* transcript levels among *A. millepora* morphs are due to gene dosage effects produced by variations in the number of highly expressed *indel (−) amilFP597* copies, representing a form of copy number polymorphism (Fig. S9, Supporting information). However, at present, it is not possible to conclude whether the differences in the maximal RFP expression levels of the morphs are functionally related to the presence of the indels or due to differences elsewhere in the gene. Alternatively, the *amilFP597* copies might constitute different allelic states of the same gene cluster in which the frequency of the *indel (−)* promoter variant defines the RFP expression level of a particular colour morph. These gene clusters could be the product of multiple tandem duplications of *amilFP597*.

As the light‐driven regulation of the expressing *amilFP597* copies in *A. millepora* translates into a pronounced phenotypic plasticity, the colour morphs should be considered the result of interacting effects of polymorphisms and polyphenisms.

The *indel (+)* and the *indel (−)* promoter variants show equally strong promoter activity in heterologous reporter gene assays (Fig. S5, Supporting information). This may indicate that the low expression of the *indel (+)*‐containing *amilFP597* copies in the adult coral is the result of gene silencing or tight repression rather than a complete loss of gene function. The completion of the *A. millepora* genome, which is expected in the near future, promises further insight into the genomic basis of colour polymorphisms in reef corals.

### Implications of colour polymorphism for photoprotection

**Figure 5:**
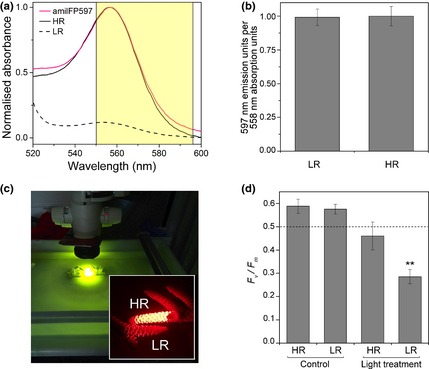
Effects of high‐level expression of amilFP597 on zooxanthellae exposed to light stress. (a) Quantitative differences in the absorption spectra of clarified tissue extracts of the high‐level red (HR) and low‐level red (LR) *Acropora millepora* morphs. The spectra show maxima at 558 nm, the amilFP597 absorption/excitation maximum. The absorption spectrum of purified recombinant amilFP597 normalized to the peak value of the HR spectrum is included for comparison. The spectral bandwidth of the light source used for stress treatments is indicated by the yellow‐shaded area. (b) Identical fluorescence emission values at 597 nm of the tissue extracts of the HR and LR morphs result from the normalization to the absorption values at 558 nm from panel (a) and demonstrate that the absorption properties of the HR and LR morph tissues in this spectral window are dominated by the concentration of amilFP597. Bars represent means of replicate measurements, error bars show standard deviation. (c) Experimental exposure of the upper side of branches of the HR and LR morphs to local high‐intensity irradiation with narrow‐band yellow‐orange light in a flow‐through aquarium. The inset image shows the fluorescence of the experimental specimen photographed through a long‐pass red filter (>600 nm). (d) Maximum quantum yield (*F*_v_/*F*_m_) of zooxanthellae photosynthesis in the light‐exposed areas of the HR and the LR morphs was recorded as an indicator of photodamage after 3 days of treatment and 14‐h dark recovery. Zooxanthellae in branch parts outside the high‐light field were used as controls. Bars represent the means of replicate measurements, error bars show standard deviation. Asterisks indicate a significant difference in the *F*_v_/*F*_m_ between HR and LR morphs exposed to the light treatment (*t*‐test with two independent samples *P *<* *0.001).

We tested whether an elevated amilFP597 concentration could contribute to increased light stress tolerance as previously described for nonfluorescent CPs (Smith *et al*. 2013). Within the maximal absorption range of CPs (562–586 nm), these pigments were found to reduce the amount of light available to zooxanthellae by up to 50%; whereas over a wider wavelength range of photosynthetically active radiation (400–620 nm), CP‐mediated screening accounted for light reduction of up to 18% (Smith *et al*. 2013). The substantial effect of screening of wavelengths outside the major absorption bands of the photosynthetic pigment results from the light‐use efficiency of zooxanthellae, which is less spectrally discriminating than the absorption spectra of diluted pigment solutions would suggest (Smith *et al*. 2013). With an absorption/excitation maximum at 558 nm, amilFP597 could potentially perform a screening function similar to the CPs. Due to their pronounced differences in amilFP597 content, we selected the HR and LR morph for a light stress experiment. Spectroscopic measurements of tissue extracts of the two morphs revealed that the absorption properties in the relevant spectral window are indeed dominated by the concentration of amilFP597 and not by CPs that might have been undetected in previous fluorescence measurements (Fig. 5a,b). The pigmented upper side of nubbins of the two morphs was subjected to local high‐intensity irradiation with narrow‐band yellow‐orange light (Fig. 5c). After 3 days, we measured the reduction in the maximum quantum yield (*F*_v_/*F*_m_) of zooxanthellae photosynthesis after a 14‐h dark recovery, as an indicator of photodamage. The zooxanthellae in branch parts outside the high‐light field were used as controls. In agreement with the results obtained for CP‐containing *Acropora valida* (Smith *et al*. 2013), the zooxanthellae of the HR morph of *A. millepora* showed less photodamage in the irradiated sites compared to those of the LR morph (Fig. 5d). The control areas of both morphs remained unaffected by the treatment. This result suggests that RFPs can exert a sun screening function in the yellow‐orange spectral range comparable to CPs. Moreover, these data support a role for fluorescence in photoprotection in shallow water by the dissipation of excess light energy via re‐emission at longer wavelengths (Wiedenmann *et al*. 1999; Salih *et al*. 2000; Dove *et al*. 2001). We note, however, that these findings do not rule out that RFPs might fulfil other or additional biological functions in coral tissue.

### Ecological implications of colour polymorphism

High sequence similarity is observed among *amilFP597* promoter fragments (Fig. S6, Supporting information) within and between the different colour morphs, suggesting that the genes are highly conserved rather than being subject to random gene loss or conversion into pseudogenes. The maintenance of different alleles/copies featuring multiple high‐ and low‐expressing *amilFP597* promoter variants might be the product of balancing selection. This process could result from variable fitness of the colour morphs over the habitat range where high‐level RFP expression might be either an advantage or a disadvantage. The production of the individual pigment molecule can be considered relatively cheap due to the low turnover rate in the tissue and the fact that only a single gene is required to yield the functional product (Leutenegger *et al*. 2007b). However, our data show that multiple copies of the relevant gene are required to achieve the previously observed high‐level expression (Leutenegger *et al*. 2007b; Oswald *et al*. 2007). Thus, maintaining high pigment concentrations represents a significant energy investment that may only be a selective advantage for individuals that occupy habitats in which light stress exceeds critical thresholds on a regular basis or during occasional episodes. On the other hand, low‐level pigment production could be advantageous for individuals from habitats with reduced light stress, as the conserved resources might be invested in higher reproductive output or faster growth. The differential expression of multiple gene copies is an ideal solution to meet these contrasting ecological demands at the population level, with the capacity for light‐driven regulation enabling further fine tuning of pigment production at the level of the individual.

Colour morphs resulting from high‐level expression of differently coloured GFP‐like proteins are not restricted to scleractinian corals, but are frequently found among other anthozoan taxa (Wiedenmann *et al*. 1999; Kelmanson & Matz 2003; Leutenegger *et al*. 2007a; Schnitzler *et al*. 2008). Their pigments are rather remotely related (Alieva *et al*. 2008), suggesting that colour polymorphism has evolved multiple times in the different lineages and may represent a common adaptation strategy. Not all anthozoan GFP‐like protein genes are regulated by light (Leutenegger *et al*. 2007b; Vogt *et al*. 2008). Consequently, some colour morphs might be defined solely by gene copy number variations or the abundance of expressed alleles (Fig. S7, Supporting information).

The findings of our study reveal the genomic basis for a novel adaptation strategy that allows individual representatives of a coral species to adjust to changing light levels along a steep environmental gradient. Moreover, as copy number polymorphisms are often found among environmentally regulated genes (Korbel *et al*. 2008), this genomic framework may explain why some individuals show the transcriptional frontloading of stress‐response genes previously demonstrated for heat‐tolerant corals (Barshis *et al*. 2013) and the large intraspecific differences in transcript levels of numerous *A. millepora* genes (Granados‐Cifuentes *et al*. 2013). Finally, this strategy might enable coral populations to adapt rapidly to long‐term changes in environmental conditions.

In conclusion, the results of this study provide a roadmap to further our understanding of stress resistance in corals, an important prerequisite to predict the capacity of coral reefs to survive under the pressure of a changing environment.

C.D. and J.W. introduced the research question. J.R.G., C.D. and J.W. designed the study, performed the research and wrote the manuscript. F.O. tested promoter activities. R.E. analysed genomic data. All authors discussed the results, revised and approved the paper.

## Data accessibility

DNA sequences:

GenBank Accession nos.: JQ009183, KC818413, KC818414, EZ013771, AAT77753.1, KJ729554, KM101115–KM101116.

The following data sets:

Absorption spectra, amilFP597 copies quantification data, all sequences of amilFP597 i*ndel (+)* and *indel (−)* promoter variants, all sequences of amilFP597 exon 3, amilFP597 promoter quantification data, spectroscopic characteristics of GFP‐like protein and all sequences utilized to reconstruct the RFP full‐length gene have been deposited in Dryad Digital Repository (http://doi.org/10.5061/dryad.5d079).
Alignments of genomic exon 3 regions of amilFP597 and paralogues and amino acid sequences of amilFP597, amilCP506 and amilCP564 are supplied in the Supporting Information.

## Supporting information


**Fig. S1** Colour polymorphism among *Acropora* sp. in Florence Bay, Magnetic Island, Great Barrier Reef, Australia.
**Fig. S2** Time course of changes in the amilFP597 tissue content of the *A. millepora* HR morph during acclimatization following altered light exposure.
**Fig. S3** Control experiment to demonstrate the suitability of semi‐quantitative *A. millepora* genome.
**Fig. S4** Red fluorescence emission spectra of the LR, MR and HR morphs and purified recombinant amilFP597.
**Fig. S5** Activity of the *amilFP597* promoter variants determined in a heterologous luciferase reporter gene assay.
**Fig. S6** Analyses of *amilFP597 indel (+)* and *indel (−)* promoter variants in different *A. millepora* colour morphs.
**Fig. S7** Analysis of genomic exon 3 regions of *amilFP597* and paralogues.
**Fig. S8** Characterisation of amilFP597 and its variants amilCP506 and amilCP564.
**Fig. S9** Conceptual model of the genomic basis of red colour polymorphism in *Acropora millepora*.
**Table S1** Oligonucleotide primers used in this study.
**Table S2** Spectroscopic characteristics of GFP‐like proteins.
