# Supplementary material for: Fluorescent protein‐mediated colour polymorphism in reef corals: multicopy genes extend the adaptation/acclimatization potential to variable light environments
Source: Mol Ecol. 2015 Jan 16;24(2):453–65. doi: 10.1111/mec.13041 (PMC4949654; doi:10.1111/mec.13041)
Supplement: Supplementary file 1 — Fig. S1 Colour polymorphism among Acropora sp. in Florence Bay, Magnetic Island, Great Barrier Reef, Australia. Fig. S2 Time course of changes in the amilFP597 tissue content of the A. millepora HR morph during acclimatization following altered light exposure. Fig. S3 Control experiment to demonstrate the suitability of semi‐quantitative A. millepora genome. Fig. S4 Red fluorescence emission spectra of the LR, MR and HR morphs and purified recombinant amilFP597. Fig. S5 Activity of the amilFP597 promoter variants determined in a heterologous luciferase reporter gene assay. Fig. S6 Analyses of amilFP597 indel (+) and indel (−) promoter variants in different A. millepora colour morphs. Fig. S7 Analysis of genomic exon 3 regions of amilFP597 and paralogues. Fig. S8 Characterisation of amilFP597 and its variants amilCP506 and amilCP564. Fig. S9 Conceptual model of the genomic basis of red colour polymorphism in Acropora millepora. Table S1 Oligonucleotide primers used in this study. Table S2 Spectroscopic characteristics of GFP‐like proteins. [file MEC-24-453-s001.pdf]

Supporting Information for online publication

Fluorescent protein-mediated colour polymorphism in reef corals: Multi-copy genes extend the adaptation/acclimatization potential to variable light environments

John R. Gittins[1], Cecilia D'Angelo[1], Franz Oswald[2], Richard J. Edwards[3,4,5] and Jörg Wiedenmann[1,5,6]

[1] Coral Reef Laboratory, Ocean and Earth Science, University of Southampton, Waterfront Campus, National Oceanography Centre, Southampton, SO14 3ZH, UK.

[2] Department of Internal Medicine I, University Medical Center Ulm, 89081 Ulm, Germany.

[3] School of Biotechnology and Biomolecular Sciences, The University of New South Wales, Sydney NSW 2052, Australia.

[4] Centre for Biological Sciences, University of Southampton, Highfield Campus, Southampton, SO171BJ, UK.

[5] Institute for Life Sciences, University of Southampton, Highfield Campus, Southampton, SO171BJ, UK.

[6] Corresponding author: Prof. Dr. Jörg Wiedenmann
Ocean and Earth Science
University of Southampton
Waterfront Campus
National Oceanography Centre
Southampton, SO14 3ZH, UK
Fax (+44) 023 8059 3059
joerg.wiedenmann@noc.soton.ac.uk

Supporting Materials and Methods

Accumulation and turnover of amilFP597 in response to changes in light exposure

Horizontally growing branches (~5 cm length) of the HR morph of *A. millepora* were removed from the mother colony, turned over and attached to stands so that the previously shaded underside became the upper side and vice versa. The decrease in red tissue fluorescence in the "new" underside of the branches was recorded throughout the acclimatization period using a fibre optic probe coupled to a Cary Eclipse fluorescence spectrometer (Varian) (D'Angelo et al. 2008). Data showing the increase in red fluorescence in the newly light-exposed branch surface, recorded in the same manner during a previous study (Hume et al. 2013), were used for comparison. The acclimatization of the tissue to the new light conditions, with regard to the amilFP597 content, was completed within ~6 weeks (Supporting Fig. S2).

Analysis of the genomic linker region between *amilFP597* copies within tandem gene arrays

The linker regions between tandem *amilFP597* copies in the *A. millepora* genome were amplified using a nested PCR approach. The primers RFP_tandem_F (binding in exon 5) and RFP_tandem_R (binding in the proximal promoter region) were used in PCRs with 0.5 µg of gDNA of the HR, MR or LR morphs of *A. millepora* as template. We introduced 2 µl of a ten-fold dilution of the PCR products as template in secondary PCRs using the nested primers RFP_tandem_F2 and RFP_tandem_R2. A 3-kb fragment dominated the amplified products of the studied morphs. This band was gel purified from the PCR product of the MR morph and cloned as described in the Materials and Methods section of the main document. Two separate 3-kb fragment clones were fully sequenced using vector and internal sequencing primers. The sequences were submitted to GenBank (acc. nos. KM101115 and KM101116).

Promoter activity of *amilFP597* variants examined using a heterologous luciferase reporter gene assay

<u>Vector construction</u>**:** DNA fragments (~1.1 kb) representing the *indel (-)* and *indel (+)* forms of the *amilFP597* promoter were separately inserted into the pGL3-Basic reporter vector (Promega, USA) upstream of the luciferase coding sequence. The reporter construct pGL3-Control (Promega, USA), in which the luciferase expression is driven by the strong Simian Virus 40 (SV40) promoter, was used as a positive control. The pGL3-Basic vector lacking promoter sequences was included as a negative control.

<u>Cell culture and transfection</u>**:** HeLa cells (ATCC CCL 2) were grown in Dulbecco's modified Eagle's medium (DMEM, Gibco, Life Technologies, USA) supplemented with 10% fetal calf serum (FCS), penicillin and streptomycin at 37°C in a 5% CO_2 atmosphere. Cells were transfected with purified plasmid DNA using Nanofectin transfection reagent (PAA, GE Healthcare, USA) according to the manufacturer's instructions.

<u>Luciferase assay</u>**:** Promoter activity was determined as described previously (Oswald et al. 2002). Briefly, HeLa cells (5 x 10^4) were transfected in 24-well plates with various amounts of reporter vector DNA to determine optimal assay conditions. The cells were harvested 24 h

after transfection, mixed with 120 µl lysis buffer [25 mM Tris/HCl (pH 7.8), 2 mM DTT, 2 mM EDTA, 1% Triton X-100, 10% glycerol] and held at room temperature for 10 min. Luciferase activity in 20 µl aliquots of the cleared lysates (7000 g, 5 min) was determined using the luciferase assay system (Promega, USA) with a Lumat LB 9501 luminometer (Berthold, Germany). The quantitative assays were performed using 0.5 µg of vector DNA for transfection. Activity values from at least four independent transfections were normalised to the level of protein and means with standard deviations were calculated.

Control experiment to validate the semi-quantitative PCR and cloning approach used to determine the relative abundance of *amilFP597* variants in the *A. millepora* genome

The primers RFPex3consF/RFPex3consR, designed to amplify a 155-bp fragment of genomic DNA spanning the chromophore coding sequence in exon 3 of *amilFP597* and its variants, were used in a control experiment to ensure the uniform amplification behaviour of this fragment from paralogous variants in semi-quantitative PCR. Linearised plasmids (1000 copies) containing the cDNAs of *amilFP597*, *amilCP506* and *amilCP564*, were used as templates for separate PCRs run using the conditions employed for *A. millepora* gDNA templates. At the end of cycles 27, 28, 29 and 30, three replicate reactions per template were removed from the thermal cycler. Agarose gel electrophoresis of the PCR products showed an essentially identical amplification profile for each 155-bp amplicon up to cycle 29 (Supporting Fig. S3). The fragments amplified from each of the triplicate reactions run for 29 cycles were cloned using the protocol employed for gDNA amplicons detailed in the Materials and Method section in the main document. The mean numbers of transformant clones were comparable for each *amilFP597* variant as was the proportion of recombinants (Supporting Fig. S3), indicating that the cloning efficiency of the 155-bp amplicons is practically identical.

Supporting Results

Characterisation of paralogous variants of amilFP597

The nucleotide sequences of the cDNAs encoding four hypothetical amilFP597-related proteins showed high similarity to a larval mRNA encoding a protein with a TYG chromophore (Supporting Fig. S7, GenBank acc. no. EZ013771; Meyer et al. 2009). The chromoproteins amilCP506 and amilCP564 are characterised by high molar extinction coefficients (Supporting Table S2), which are comparable to those of CPs with light screening capacity (Smith et al. 2013). At the same time, their very low fluorescence quantum yields render them essentially non-fluorescent. The dramatic reduction in fluorescence compared to amilFP597 can be attributed to a serine to cysteine replacement in these proteins that affects the position homologous to residue 148 in amilFP597 (Supporting Fig. S8). This alteration can be expected to cause the loss of a hydrogen bond to the phenolate of the chromophore, most likely inducing its cis-trans isomerisation that produces the observed low fluorescent state (Gurskaya et al. 2001; Prescott et al. 2003; Nienhaus et al. 2008; Nienhaus & Wiedenmann 2009). In contrast, the loss of fluorescence in the *A. millepora* chromoproteins amilCP575, amilCP584 and amilCP604, which are highly expressed in some adult

A. millepora morphs (Smith et al. 2013), is associated with the replacement of serine 148 by asparagine or histidine.

Exchanges in the first position of the chromophore-forming amino acid triplet can prevent the formation of the extended π-electron system required for the absorption of yellow-orange wavelengths (Wiedenmann et al. 2004). Hence, the substitution of aspartate in the first position of this triplet in amilFP597 by threonine in amilCP506 is the likely cause of the unusual absorption maximum in the green spectral range.

As detailed in the Results and Discussion section of the manuscript, amilCP506 and amilCP564 have likely evolved from amilFP597. Their high level expression during larval stages (Beltran Ramirez 2010) together with the separate evolutionary history suggests that the amilFP597-derived CPs might have adopted new functions during the early development of *A. millepora*, such as selective screening of certain wavelengths of light (Supporting Fig. S8). This screening might fulfill photoprotective functions (Smith et al. 2013) or modulate photoreceptor responses, for example, during larval settlement. The amino acid exchanges that affect key functional residues and alter the spectral properties of the amilFP597-derived CPs contribute to a small overall number of non-conservative mutations that distinguishes them from amilFP597, suggesting that these variants are under strong positive selection, rather than being subject to random mutagenesis leading to loss of function.

Supporting References

Beltran Ramirez V (2010) Molecular aspects of the fluorescent protein homologues in *Acropora millepora*. James Cook University.

D'Angelo C, Denzel A, Vogt A, et al. (2008) Blue light regulation of host pigment in reef-building corals. *Marine Ecology Progress Series* **364**:97-106.

Gurskaya NG, Fradkov AF, Terskikh A, et al. (2001) GFP-like chromoproteins as a source of far-red fluorescent proteins. *FEBS Letters* **507**:16-20.

Hume B, D'Angelo C, Burt J, et al. (2013) Corals from the Persian/Arabian Gulf as models for thermotolerant reef-builders: Prevalence of clade C3 *Symbiodinium*, host fluorescence and *ex situ* temperature tolerance. *Marine Pollution Bulletin* **72**:313-322.

Meyer E, Aglyamova GV, Wang S, et al. (2009) Sequencing and de novo analysis of a coral larval transcriptome using 454 GSFlx. *BMC Genomics* **10**:219.

Nienhaus GU, Wiedenmann J (2009) Structure, dynamics and optical properties of fluorescent proteins: Perspectives for marker development. *Chemphyschem* **10**:1369-1379.

Nienhaus K, Nar H, Heilker R, et al. (2008) Trans-cis isomerization is responsible for the red-shifted fluorescence in variants of the red fluorescent protein eqFP611. *Journal of the American Chemical Society* **130**:12578-12579.

Oswald F, Kostezka U, Astrahantseff K, et al. (2002) SHARP is a novel component of the Notch/RBP-Jκ signalling pathway. *EMBO Journal* **21**:5417-5426.

Prescott M, Ling M, Beddoe T, et al. (2003) The 2.2 A crystal structure of a pocilloporin pigment reveals a nonplanar chromophore conformation. *Structure* **11**:275-284.

Smith EG, D'Angelo C, Salih A, Wiedenmann J (2013) Screening by coral green fluorescent protein (GFP)-like chromoproteins supports a role in photoprotection of zooxanthellae. *Coral Reefs* **32**:463-474.

Wiedenmann J, Ivanchenko S, Oswald F, et al. (2004) EosFP, a fluorescent marker protein with UV-inducible green-to-red fluorescence conversion. *Proceedings of the National Academy of Science of United States of America* **101**:15905-15910.

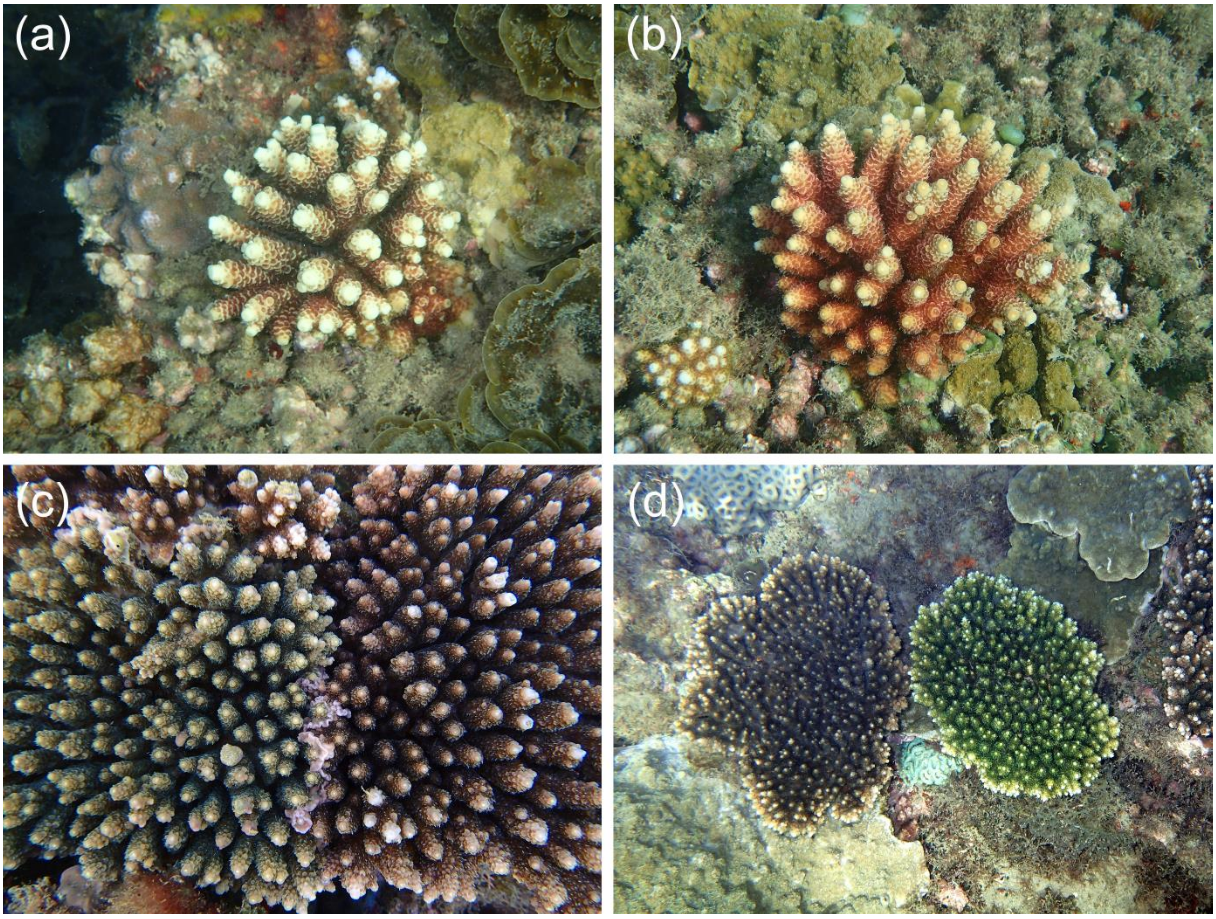

Supporting Figure S1: Colour polymorphism among *Acropora* sp. in Florence Bay, Magnetic Island, Great Barrier Reef, Australia. (a-b) A brown *A. millepora* colony (a) growing at the same depth (~1 m) and within 1 m distance from an individual (b) showing high-level RFP expression. Distinct differences in RFP pigmentation exist despite exposure to identical light levels. (c-d) Co-existence of *Acropora* sp. colonies showing pronounced differences in the expression of GFPs under the same conditions demonstrate the widespread occurrence of colour polymorphism mediated by GFP-like proteins.

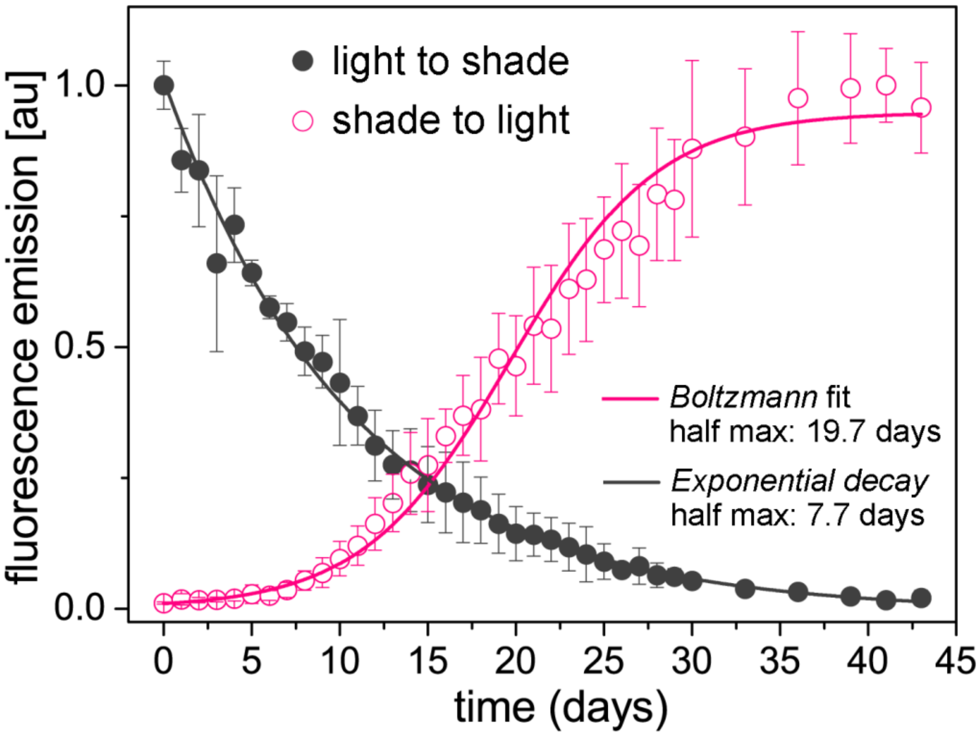

Supporting Figure S2: Time course of changes in the amilFP597 tissue content of the *A. millepora* HR morph during acclimatization following altered light exposure. The red tissue fluorescence was recorded using a fibre optic probe coupled to a fluorescence spectrometer after horizontal branches were turned over to expose the previously shaded side to light and vice versa. Data points represent means of replicate measurements and error bars give the standard deviation. The light-to-shade and shade-to-light data were fitted with an exponential decay function and a non-linear curve fit (Boltzmann), respectively. Half-maximal values of the fitted curves are indicated.

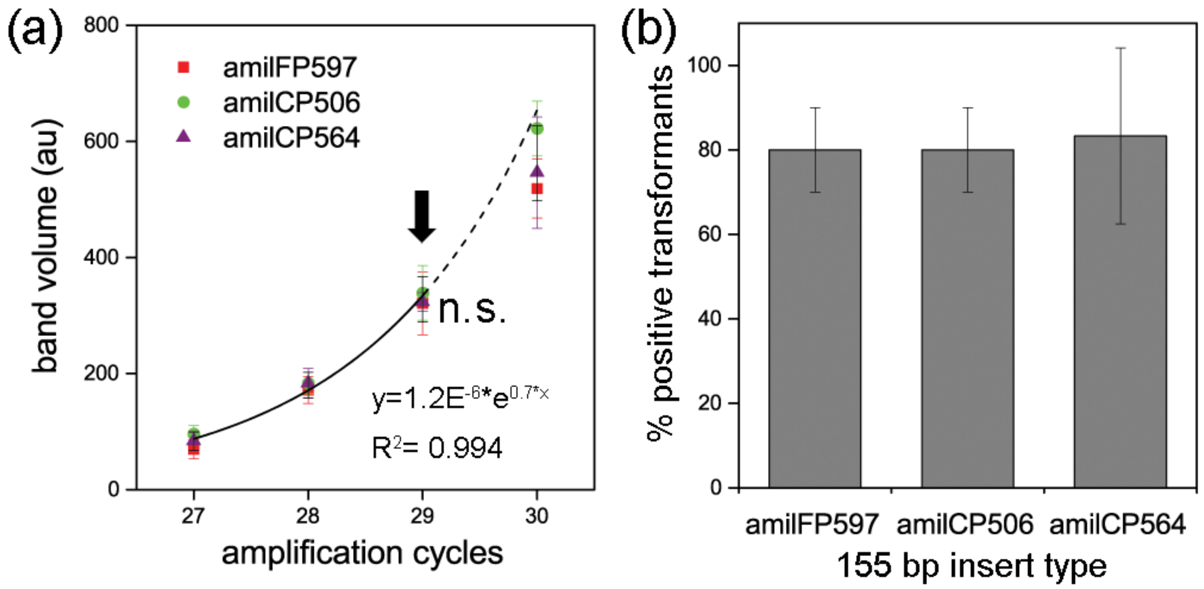

Supporting Figure S3: Control experiment to demonstrate the suitability of semi-quantitative PCR and cloning to determine the relative abundance of *amilFP597* variants in the *A. millepora* genome. (a) Uniform and comparable PCR amplification of the 155-bp exon 3 fragments of *amilFP597* variants from cDNA templates. The values plotted represent mean volumes of ethidium bromide-stained bands following agarose gel analysis of triplicate PCRs after the indicated number of cycles. Error bars indicate standard deviation. Data points representing cycles 27-29 were fitted with an exponential function (solid line). This curve was extended to the cycle 30 data (dashed line). The formula and R^2 values are shown. While the PCRs were still within the exponential amplification phase, differences between the amplification efficiencies for the three variants were not significant (n.s.) according to one way ANOVA (Probability value =0.18 for cycle 29 data). (b) The cloning efficiency of the 155-bp fragments representing different *amilFP597* variants obtained after 29 cycles of semi-quantitative PCR amplification (arrowhead) is comparable. The values represent the proportion of positive clones among 30 randomly picked colonies. Error bars indicate the standard deviation from triplicate cloning experiments.

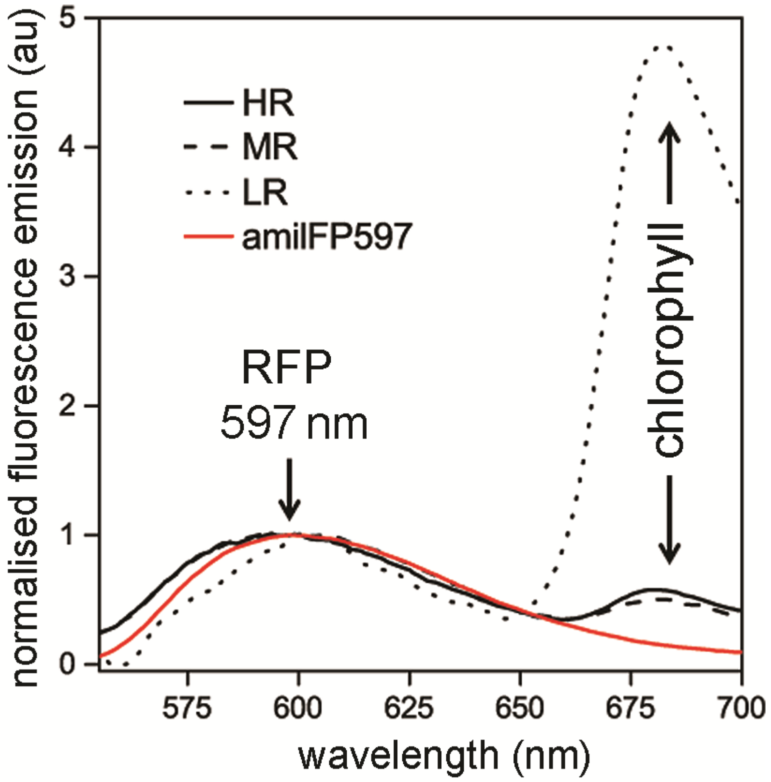

Supporting Figure S4: Red fluorescence emission spectra of the LR, MR and HR morphs and purified recombinant amilFP597. Spectra were normalised to the maximal value of the RFP fluorescence at ~597 nm. Fluorescence was excited at 530 nm. The chlorophyll fluorescence of the zooxanthellae in the coral tissue produces a maximum at ~683 nm.

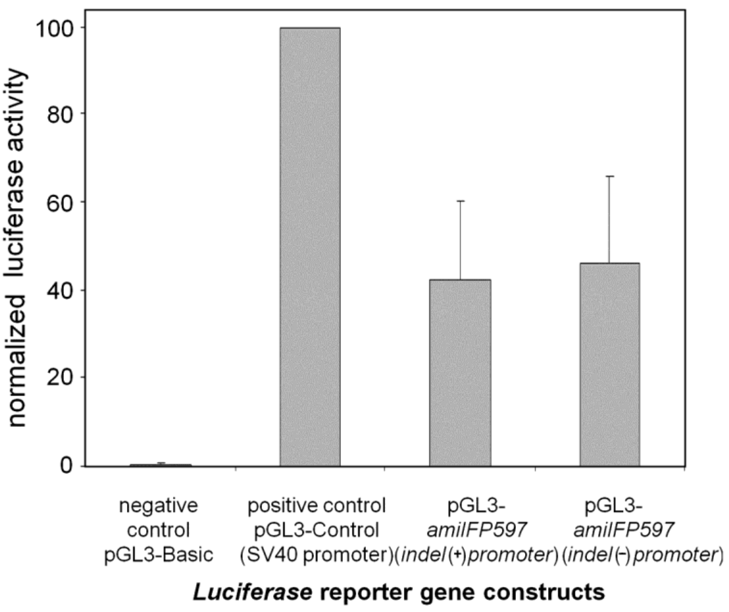

Supporting Figure S5: Activity of the *amilFP597* promoter variants determined in a heterologous luciferase reporter gene assay. The *indel (+)* and *indel (-)* promoter fragments including the 5'UTR were inserted upstream of the luc gene in the vector pGL3-Basic. Luciferase activity was assayed after transfection of HeLa cells. Positive (pGL3-Control, SV40 promoter-luc) and negative (pGL3-Basic) control vectors were analysed in parallel. The luciferase activity data were normalised to the total protein content of the cell lysates. The graph shows the mean normalised values from 5 independent experiments using 0.5 µg vector DNA for transfection. Error bars denote standard deviation.

(a)

Percent Identity

Divergence

	1	2	3	4	5	6	7	8	9	10	11	12	13	14	15	16	17	18	19	20	21	22	23	24	25	26	27	28	29		
1		99.5	100.0	99.8	99.7	99.8	99.7	99.6	99.8	99.8	99.5	98.6	99.0	99.1	99.8	99.5	99.7	99.1	99.1	99.0	99.0	99.1	99.5	99.1	99.0	99.1	99.0	99.1	99.1	1	indel (+) amiFP597_MR1
2	0.5		99.5	99.3	99.2	99.3	99.2	99.0	99.3	99.3	99.0	98.1	98.5	98.6	99.3	99.0	99.2	98.6	98.6	98.5	98.5	98.6	99.0	98.6	98.5	98.6	98.4	98.6	98.6	2	indel (+) amiFP597_MR2
3	0.0	0.5		99.8	99.7	99.8	99.7	99.6	99.8	99.8	99.5	98.6	99.0	99.1	99.8	99.5	99.7	99.1	99.1	99.0	99.0	99.1	99.5	99.1	99.0	99.1	99.0	99.1	99.1	3	indel (+) amiFP597_MR3
4	0.2	0.7	0.2		99.9	100.0	99.9	99.7	100.0	100.0	99.7	98.8	99.2	99.3	100.0	99.7	99.9	99.3	99.3	99.2	99.2	99.3	99.7	99.3	99.2	99.3	99.1	99.3	99.3	4	indel (+) amiFP597_LR1
5	0.3	0.8	0.3	0.1		99.9	99.8	99.7	99.9	99.9	99.6	98.7	99.1	99.2	99.9	99.6	99.8	99.2	99.2	99.1	99.1	99.2	99.6	99.2	99.1	99.2	99.0	99.2	99.2	5	indel (+) amiFP597_LR2
6	0.2	0.7	0.2	0.0	0.1		99.9	99.7	100.0	100.0	99.7	98.8	99.2	99.3	100.0	99.7	99.9	99.3	99.3	99.2	99.2	99.3	99.7	99.3	99.2	99.3	99.1	99.3	99.3	6	indel (+) amiFP597_LR3
7	0.3	0.8	0.3	0.1	0.2	0.1		99.8	99.9	99.9	99.6	98.7	99.1	99.2	99.9	99.6	99.8	99.2	99.2	99.1	99.1	99.2	99.6	99.2	99.1	99.2	99.0	99.2	99.2	7	indel (+) amiFP597_LR4
8	0.4	1.0	0.4	0.3	0.3	0.3	0.2		99.7	99.7	99.4	98.5	99.0	99.0	99.7	99.4	99.7	99.0	99.0	99.0	99.0	99.0	99.4	99.0	99.0	99.0	98.9	99.0	99.0	8	indel (+) amiFP597_LR5
9	0.2	0.7	0.2	0.0	0.1	0.0	0.1	0.3		100.0	99.7	98.8	99.2	99.3	100.0	99.7	99.9	99.3	99.3	99.2	99.2	99.3	99.7	99.3	99.2	99.3	99.1	99.3	99.3	9	indel (+) amiFP597_LR6
10	0.2	0.7	0.2	0.0	0.1	0.0	0.1	0.3	0.0		99.7	98.8	99.2	99.3	100.0	99.7	99.9	99.3	99.3	99.2	99.2	99.3	99.7	99.3	99.2	99.3	99.1	99.3	99.3	10	indel (+) amiFP597_HR1
11	0.5	1.1	0.5	0.3	0.4	0.3	0.4	0.6	0.3	0.3		98.4	99.0	99.0	99.7	99.7	99.6	99.3	99.3	99.2	99.2	99.3	99.3	99.3	99.2	99.3	99.1	99.3	99.3	11	indel (+) amiFP597_HR2
12	1.4	1.9	1.4	1.2	1.3	1.2	1.3	1.5	1.2	1.2	1.6		99.4	99.5	98.8	98.8	98.7	98.6	98.6	98.5	98.5	98.6	98.6	98.6	98.5	98.6	98.4	98.6	98.6	12	indel (+) amiFP597_IR1.1
13	1.0	1.5	1.0	0.8	0.9	0.8	0.9	1.1	0.8	0.8	1.0	0.6		99.9	99.2	99.2	99.1	99.0	99.0	99.0	99.0	99.0	99.0	99.0	99.0	99.0	98.9	99.0	99.0	13	indel (+) amiFP597_IR1.2
14	0.9	1.4	0.9	0.7	0.8	0.7	0.8	1.0	0.7	0.7	1.1	0.5	0.1		99.3	99.3	99.2	99.1	99.1	99.0	99.0	99.1	99.1	99.1	99.0	99.1	99.0	99.1	99.1	14	indel (+) amiFP597_IR1.3
15	0.2	0.7	0.2	0.0	0.1	0.0	0.1	0.3	0.0	0.0	0.3	1.2	0.8	0.7		99.7	99.9	99.3	99.3	99.2	99.2	99.3	99.7	99.3	99.2	99.3	99.1	99.3	99.3	15	indel (+) amiFP597_IR2.1
16	0.5	1.1	0.5	0.3	0.4	0.3	0.4	0.6	0.3	0.3	0.3	1.2	0.8	0.7	0.3		99.6	99.7	99.7	99.6	99.6	99.7	99.3	99.7	99.6	99.7	99.5	99.7	99.7	16	indel (+) amiFP597_IR2.2
17	0.3	0.8	0.3	0.1	0.2	0.1	0.2	0.3	0.1	0.1	0.4	1.3	0.9	0.8	0.1	0.4		99.2	99.2	99.1	99.1	99.2	99.6	99.2	99.1	99.2	99.0	99.2	99.2	17	indel (+) amiFP597_IR2.3
18	0.9	1.4	0.9	0.7	0.8	0.7	0.8	1.0	0.7	0.7	0.7	1.4	1.0	0.9	0.7	0.3	0.8		99.8	99.7	99.7	99.8	99.5	99.8	99.9	99.8	99.7	99.8	99.8	18	indel (-) amiFP597_MR1
19	0.9	1.4	0.9	0.7	0.8	0.7	0.8	1.0	0.7	0.7	0.7	1.4	1.0	0.9	0.7	0.3	0.8	0.2		99.9	99.9	100.0	99.7	100.0	99.9	100.0	99.8	100.0	100.0	19	indel (-) amiFP597_MR2
20	1.0	1.5	1.0	0.8	0.9	0.8	0.9	1.1	0.8	0.8	0.8	1.5	1.1	1.0	0.8	0.4	0.9	0.3	0.1		99.8	99.9	99.6	99.9	99.8	99.9	99.7	99.9	99.9	20	indel (-) amiFP597_MR3
21	1.0	1.5	1.0	0.8	0.9	0.8	0.9	1.1	0.8	0.8	0.8	1.5	1.1	1.0	0.8	0.4	0.9	0.3	0.1	0.2		99.9	99.6	99.9	99.8	99.9	99.7	99.9	99.9	21	indel (-) amiFP597_HR1
22	0.9	1.4	0.9	0.7	0.8	0.7	0.8	1.0	0.7	0.7	0.7	1.4	1.0	0.9	0.7	0.3	0.8	0.2	0.0	0.1	0.1		99.7	100.0	99.9	100.0	99.8	100.0	100.0	22	indel (-) amiFP597_HR2
23	0.5	1.1	0.5	0.3	0.4	0.3	0.4	0.6	0.3	0.3	0.7	1.4	1.0	0.9	0.3	0.7	0.4	0.5	0.3	0.4	0.4	0.3		99.7	99.6	99.7	99.5	99.7	99.7	23	indel (-) amiFP597_HR3
24	0.9	1.4	0.9	0.7	0.8	0.7	0.8	1.0	0.7	0.7	0.7	1.4	1.0	0.9	0.7	0.3	0.8	0.2	0.0	0.1	0.1	0.0	0.3		99.9	100.0	99.8	100.0	100.0	24	indel (-) amiFP597_IR1.1
25	1.0	1.5	1.0	0.8	0.9	0.8	0.9	1.1	0.8	0.8	0.8	1.5	1.1	1.0	0.8	0.4	0.9	0.1	0.1	0.2	0.2	0.1	0.4	0.1		99.9	99.7	99.9	99.9	25	indel (-) amiFP597_IR1.2
26	0.9	1.4	0.9	0.7	0.8	0.7	0.8	1.0	0.7	0.7	0.7	1.4	1.0	0.9	0.7	0.3	0.8	0.2	0.0	0.1	0.1	0.0	0.3	0.0	0.1		99.8	100.0	100.0	26	indel (-) amiFP597_IR1.3
27	1.1	1.6	1.1	0.9	1.0	0.9	1.0	1.1	0.9	0.9	0.9	1.6	1.1	1.1	0.9	0.5	1.0	0.3	0.2	0.3	0.3	0.2	0.5	0.2	0.3	0.2		99.8	99.8	27	indel (-) amiFP597_IR2.1
28	0.9	1.4	0.9	0.7	0.8	0.7	0.8	1.0	0.7	0.7	0.7	1.4	1.0	0.9	0.7	0.3	0.8	0.2	0.0	0.1	0.1	0.0	0.3	0.0	0.1	0.0	0.2		100.0	28	indel (-) amiFP597_IR2.2
29	0.9	1.4	0.9	0.7	0.8	0.7	0.8	1.0	0.7	0.7	0.7	1.4	1.0	0.9	0.7	0.3	0.8	0.2	0.0	0.1	0.1	0.0	0.3	0.0	0.1	0.0	0.2	0.0		29	indel (-) amiFP597_IR2.3
	1	2	3	4	5	6	7	8	9	10	11	12	13	14	15	16	17	18	19	20	21	22	23	24	25	26	27	28	29		

(b)

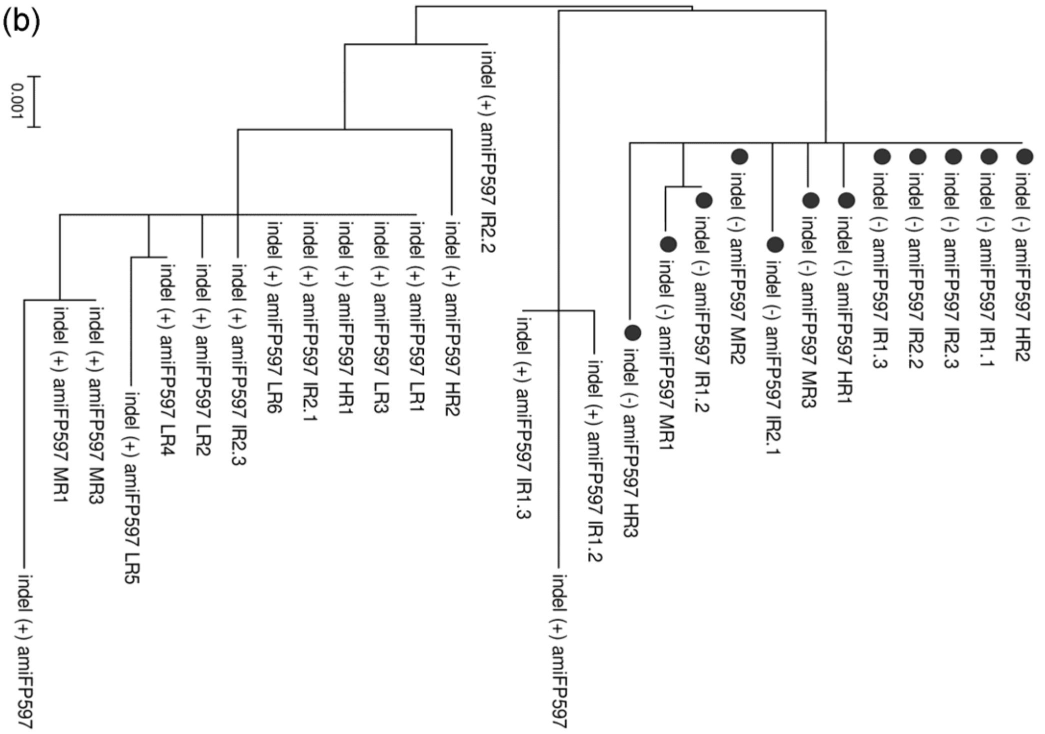

Supporting Figure S6: Analyses of *amilFP597 indel (+)* and *indel (-)* promoter variants in different *A. millepora* colour morphs. Fragments extending 1.1 kb upstream of the *amilFP597* start codon were amplified from gDNA templates by PCR using the specific primers AmRFPp-F3/AmRFPp-R1. The amplified fragments were cloned and at least 5 clones per morph sequenced. Indel regions were not included in the subsequent analyses. (a) Divergence/identity of all sequences of the two *amilFP597* promoter variants identified in the *A. millepora* HR, MR and LR morphs and morphs showing intermediate redness (IR1, IR2). (b) Midpoint rooted Maximum Likelihood molecular phylogeny inferred from all *amilFP597* promoter sequences. The tree is drawn to scale, with branch lengths measured in the number of substitutions per site. Filled black spheres highlight the *indel (-)* variants clustering together despite their origin from different colour morphs.

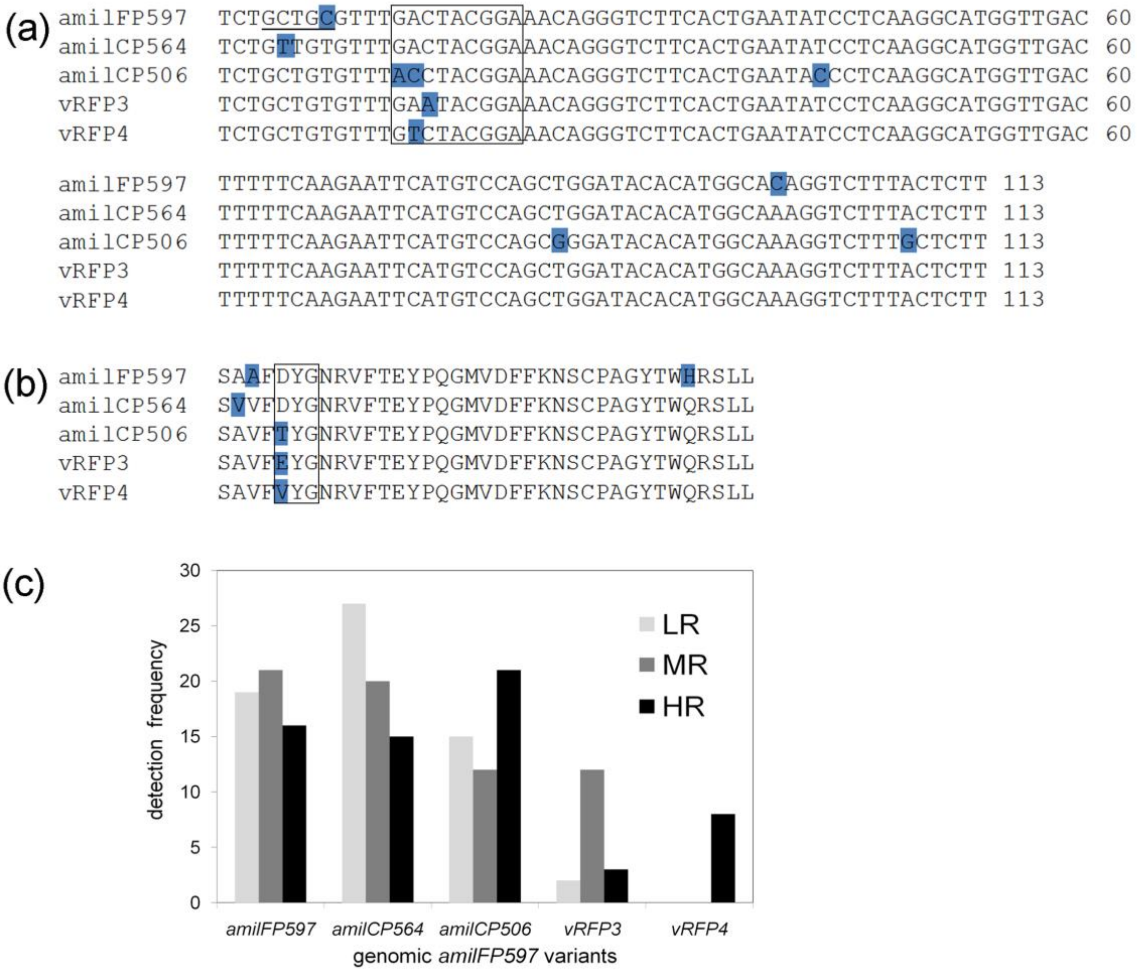

Supporting Figure S7: Analysis of genomic exon 3 regions of *amilFP597* and paralogues. (a) Multiple sequence alignment of PCR-amplified exon 3 fragments of all identified *amilFP597* variants (primer sequences removed). Besides the variants encoding amilFP597, amilCP564 and amilCP506, two further variants were identified in the *A. millepora* genome. Their transcripts could not be recovered for functional analysis of the gene product, hence they are described as “variant RFP” (vRFP) 3 and 4. Nucleotides that are different from the consensus are highlighted by blue shading. The diagnostic ApeKI site used for restriction analysis is underlined. The region encoding the chromophore-forming amino acid triplet is boxed. (b) Multiple alignment of amino acid sequences obtained by translation of the exon 3 sequences in (a). In the case of the characterised proteins, this region extends from residue 62 to 98. Amino acids that are different from the consensus are highlighted by blue shading. The chromophore triplet is boxed. (c) Detection frequency of *amilFP597* variants in different colour morphs of *A. millepora*. In the LR and HR morphs, clones representing *amilFP597* were found ~8 times more frequently than vRFP3. This variant RFP must be encoded by at least a single copy gene, suggesting that *amilFP597* is present in multiple (≥8) copies in the *A. millepora* genome. The paralogous *amilFP597* variants vRFP3 and 4 show a variable detection frequency among different morphs. The respective transcripts were not found in our adult colonies, so these variations in the absolute gene copy number are presumably not responsible for colour polymorphisms in adult *A. millepora*. However, such variations in the absolute gene copy number could be responsible for colour polymorphisms among cnidarians when the involved genes are expressed at sufficient levels.

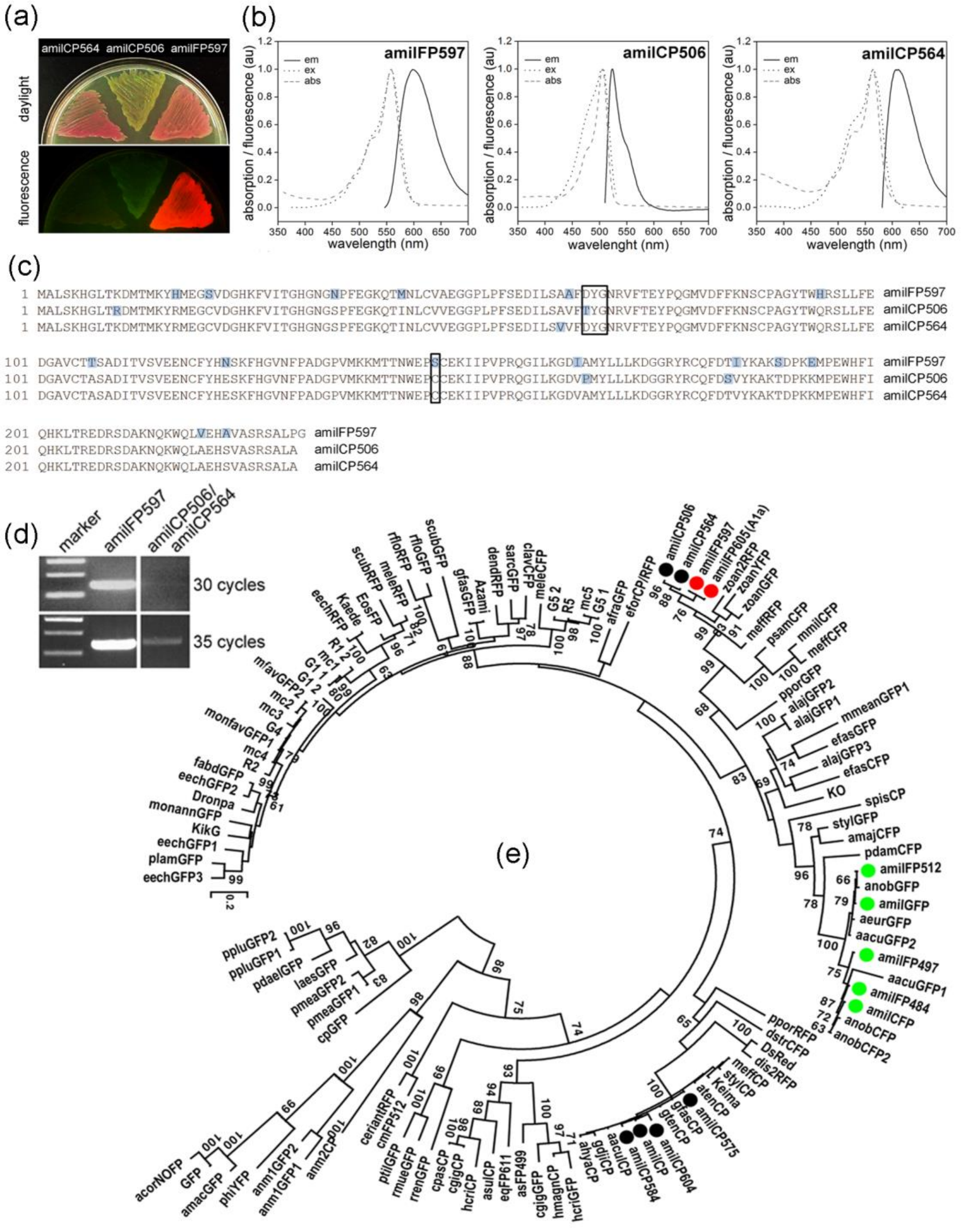

Supporting Figure S8: Characterisation of amilFP597 and its variants amilCP506 and amilCP564. (a) Photographs of *Escherichia coli* strains expressing the recombinant *A. millepora* protein pigments grown on LB-agar medium (upper panel: daylight image). Fluorescence (lower panel) was documented using BlueStar lamps for excitation and VG1 filter glasses (Nightsea, Bedford, MA, USA) mounted in front of the camera. (b) Absorption, excitation and emission spectra of purified recombinant amilFP597, amilCP506 and amilCP564. (c) Amino acid sequence alignment of amilFP597 variants encoded by cDNAs cloned from adult *A. millepora* (MR morph). Unique residues are shaded blue. The chromophore-forming triplet (residues 66-68) and residue 148 that interacts with the phenolate sidechain of the chromophore are boxed. (d) PCR-amplification of transcripts coding for amilFP597 and amilCP506/amilCP564 from a cDNA library prepared from adult

A. millepora tissue (MR morph). The fragments (~696 bp) were obtained after 30 or 35 cycles of amplification under the same conditions using identical template amounts. The image shows the PCR products and a DNA size marker separated on ethidium bromide-stained agarose gels. After 30 cycles, the *amilFP597* PCR was close to saturation whereas the CP amplicons were barely detectable, suggesting that the abundance of their cDNAs in the library was >100x lower than that of *amilFP597*. (e) Molecular phylogenetic analysis of GFP-like protein sequences applying the Maximum Likelihood method. The tree is drawn to scale, with branch lengths measured in the number of substitutions per site. Numbers indicate key percentage bootstrap support values. *A. millepora* pigments are denoted by filled spheres: red – amilFP597, black – CPs, green – cyan and green FPs. GFPs from arthropoda were used as outgroup.

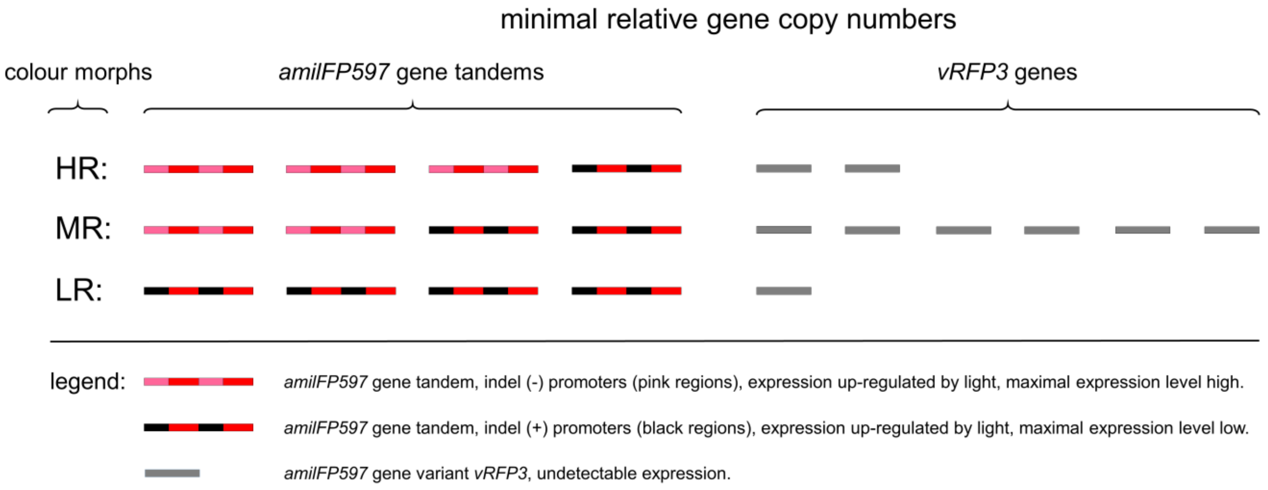

Supporting Figure S9: Conceptual model of the genomic basis of red colour polymorphism in *Acropora millepora*. The gene copy numbers encoding the red fluorescent protein amilFP597 are defined relative to the smallest copy numbers found for the *amilFP597*-paralog *vRFP3*. Minimal copy numbers are given and the real numbers might be multiples of these values. The maximal redness of the colour morphs (HR: high red; MR: medium red; LR: low red) in a specific light environment is correlated with the ratio of amilFP597 genes (arranged in tandems) that contain or lack three distinct indels (indel [+]; indel [-]) in their promoter regions. The expression of the *amilFP597* genes is up-regulated by light. The redness of individual coral colonies depends therefore on the number of *amilFP597* genes that can be expressed at high levels and the light exposure in the given habitat.

Table S1: Oligonucleotide primers used in this study

Primer Name	Primer sequence	*Binding region*
**amilFP RT-PCR**		
amilFP597F	TTTGGATCCATGGCTCTGTCAAAGCACGGTTTAAC	*amilFP597* gene
amilFP597R	TTTAAGCTTATCCGGGCAATGCGGATCGG	*amilFP597* gene
**Housekeeping RT-PCR**		
actin2_new_For	GTCGCTCTTGACTTCGAACAAG	*A. millepora actin2* gene
actin2_new_Rev	GAAGCGCGATATTTCGTCTTAG	*A. millepora actin2* gene
**GenomeWalker gene-specific**		
RFP SP1	AGTCAACCATGCCTTGAGGATATTCAG	*RFP* exon 3
RFP SP2	AGACCCTTCCATGTGGTATTTCATCG	*RFP* exon 2
**amilFP597 RACE**		
RFP 5' RACE	AGTCAACCATGCCTTGAGGATATTCAG	*RFP* gene
RFP 3' RACE	TCTGCTGCGTTTGACTACGGAAACAG	*RFP* gene
***indel (+)* or *indel (-) amilFP597* promoter**		
pRFPlargeF	GAACTCTGGAAAACTAAGGGAAATCAC	*amilFP597* promoter
pRFPsmallF	GAACTCTGGAAAACTAAGGGAAAGTCT	*amilFP597* promoter
**3' genomic region amplification**		
RFP_I2-3'U_F	ATGTGCATCAAAACCTAAGCCGTTAAG	*RFP* intron 2
RFP_I2-3'U_R2	GGCTCGAACTACGTTGTCATCAGAGTA	*RFP* 3' UTR
**Genomic linker region between *amilFP597* copies**		
RFP_tandem_F	ATCCAACATAAGCTCACCCGGGAAGAC	*amilFP597* exon 5
RFP_tandem_R	CTGAATGTAGTGTTAGGGTGGAGCAAT	*amilFP597* prox. promoter
RFP_tandem_F2	ACCAGAAATGGCAACTGGTAGAACATGC	*amilFP597* exon 5
RFP_tandem_R2	CTTGACTCGATGGGAATATATCCATGTGA	*amilFP597* prox. promoter
**RFP exon3 consensus sequence**		
RFPex3consF	GCCATTCTCCGAAGACATTTTG	*RFP* exon 3
RFPex3consR	TGCAAACTGCTCCATCTTCA	*RFP* exon 3
**Proximal promoter amplification**		
AmRFPp-F3	AGGCTATTCGGAAGGCATGA	*amilFP597* promoter
AmRFPp-R1	CTTTGACAGAGCCATGCTGA	*amilFP597* 5' UTR-exon1
**Full-length ORF amplification***		
14_28cDNA_F	TTTGGATCCATGGCTCTGTCAAAGCACGGTCTAAC	*amilFP597/amilCP506* start codon
amilFP597R	TTTAAGCTTATCCGGGCAATGCGGATCGG	*amilFP597* stop codon
14_28cDNA_R	TTTAAGCTTTGAACTATATCATGTTCTTATCAGGC	*amilCP506*/564 stop codon
**Promoter-luc reporter constructs**		
pRFPATGNco	ACAGAGCCATGGTGAGAACAGATCGCTTGA	*amilFP597* start codon

*Nucleotides added or changed to introduce restriction endonuclease cleavage sites are underlined

Table S2: Spectroscopic characteristics of GFP-like proteins

Protein	Absorption / Excitation Maximum [nm]	Emission Maximum [nm]	Quantum Yield [QY]	E_{mol} [M^{-1} cm^{-1}]	Relative Brightness [a]
eqFP611 [d]	559	611	0.45	116000	1.00 [b]
amilFP597	558	597	0.46	97000	0.85 [b]
amilFP605 (A1a [e])	559	605	0.46	116000	1.02 [b]
amilCP564	564	599	0.02	98000	0.04 [b]
EosFP [f]	506	516	0.70	72000	1.00 [c]
amilCP506	506	516	0.03	114000	0.06 [c]

[a] expressed as QY* E_{mol}

[b] Relative to eqFP611

[c] Relative to EosFP

[d] Wiedenmann J, Schenk A, Rocker C, et al. (2002) A far-red fluorescent protein with fast maturation and reduced oligomerization tendency from *Entacmaea quadricolor* (Anthozoa, Actinaria). *Proceedings of the National Academy of Sciences of the United States of America* **99**:11646-11651.

[e] The construct encoding the putative protein A1a (Smith-Keune C & Dove S, 2007. Gene expression of a green fluorescent protein homolog as a host-specific biomarker of heat stress within a reef-building coral. *Marine Biotechnology* **10**: 1-15) was created by mutagenesis of an *amilFP597* cDNA (GenBank acc. no. JX258844) to produce a synthetic A1a cDNA (GenBank acc. no. KJ729554), encoding a protein with amino acid changes N34D and M44I.

[f] Wiedenmann J, Ivanchenko S, Oswald F, et al. (2004) EosFP, a fluorescent marker protein with UV-inducible green-to-red fluorescence conversion. *Proceedings of the National Academy of Sciences of the United States of America* **101**:15905-15910.

.
